# Panethnic Differences in Blood Pressure in Europe: A Systematic Review and Meta-Analysis

**DOI:** 10.1371/journal.pone.0147601

**Published:** 2016-01-25

**Authors:** Pietro Amedeo Modesti, Gianpaolo Reboldi, Francesco P. Cappuccio, Charles Agyemang, Giuseppe Remuzzi, Stefano Rapi, Eleonora Perruolo, Gianfranco Parati, ESH Working Group on CV Risk in Low Resource Settings

**Affiliations:** 1 Dept of Medicina Sperimentale e Clinica, University of Florence, Florence, Italy; 2 Dept of Medicine, University of Perugia, Perugia, Italy; 3 University of Warwick, Warwick Medical School, and University Hospitals Coventry & Warwickshire NHS Trust, Coventry, United Kingdom; 4 Dept of Public Health, Academic Medical Centre, University of Amsterdam, Amsterdam, Netherlands; 5 IRCCS - Istituto di Ricerche Farmacologiche Mario Negri, Bergamo, Dept. of Medicine, Unit of Nephrology, Dialysis and Transplantation, Azienda Ospedaliera Papa Giovanni XXIII, Bergamo, Italy; 6 Dept of Health Sciences, University of Milano-Bicocca, Dept. of Cardiology, S. Luca Hospital, IRCCS Istituto Auxologico, Milano, Italy; Hospital de Clínicas de Porto Alegre, BRAZIL

## Abstract

**Background:**

People of Sub Saharan Africa (SSA) and South Asians(SA) ethnic minorities living in Europe have higher risk of stroke than native Europeans(EU). Study objective is to provide an assessment of gender specific absolute differences in office systolic(SBP) and diastolic(DBP) blood pressure(BP) levels between SSA, SA, and EU.

**Methods and Findings:**

We performed a systematic review and meta-analysis of observational studies conducted in Europe that examined BP in non-selected adult SSA, SA and EU subjects. Medline, PubMed, Embase, Web of Science, and Scopus were searched from their inception through January 31st 2015, for relevant articles. Outcome measures were mean SBP and DBP differences between minorities and EU, using a random effects model and tested for heterogeneity. Twenty-one studies involving 9,070 SSA, 18,421 SA, and 130,380 EU were included. Compared with EU, SSA had higher values of both SBP (3.38 mmHg, 95% CI 1.28 to 5.48 mmHg; and 6.00 mmHg, 95% CI 2.22 to 9.78 in men and women respectively) and DBP (3.29 mmHg, 95% CI 1.80 to 4.78; 5.35 mmHg, 95% CI 3.04 to 7.66). SA had lower SBP than EU(-4.57 mmHg, 95% CI -6.20 to -2.93; -2.97 mmHg, 95% CI -5.45 to -0.49) but similar DBP values. Meta-analysis by subgroup showed that SA originating from countries where Islam is the main religion had lower SBP and DBP values than EU. In multivariate meta-regression analyses, SBP difference between minorities and EU populations, was influenced by panethnicity and diabetes prevalence.

**Conclusions:**

1) The higher BP in SSA is maintained over decades, suggesting limited efficacy of prevention strategies in such group in Europe;2) The lower BP in Muslim populations suggests that yet untapped lifestyle and behavioral habits may reveal advantages towards the development of hypertension;3) The additive effect of diabetes, emphasizes the need of new strategies for the control of hypertension in groups at high prevalence of diabetes.

## Introduction

The high rate of influx and settlement of migrant populations into Europe is changing the dynamics of regional population growth, and may pose important challenges for public health and clinical care [1]. Both subjects originating from Sub Saharan Africa (SSA) [2] and South Asia (SA) [3] were found to have higher risk of stroke [4,5] and end-stage renal failure[5,6] than native Europeans(EU). SA subjects living in Europe also have elevated risk of coronary heart disease [5,7,8]. Understanding the reasons behind the excess of cardiovascular (CV) risks is crucial for addressing ethnic inequalities in health also because the ageing of migrant populations carries the risk of overburdening the majority of the healthcare systems in the European Union that offer free of charge access to emergency medical care [9]. In the USA, studies on ethnic variation in health risk reveal a major confounding effect by socio-economic status [10]. In Europe, ethnic differences in CV events persist after adjusting for income differences, suggesting that other variables might play a role [11]. Detection and control of risk factors such as hypertension and diabetes, identification of potential drivers predisposing ethnic minority groups to CV disease, and recognizing at risk sub-populations may be crucial to build effective prevention strategies [12,13]. Currently only four European Union member states (Netherlands, France, Portugal and Spain) cover the costs of long-term medical care for chronic disease in undocumented migrants [1,14]. However, previous reviews collecting UK based studies data were not pooled making it difficult to estimate actual differences between the groups.

As a major CV risk factor, hypertension needs to be carefully managed. An early review showed higher blood pressure(BP) levels in SSA than in EU [15]. Reductions in salt intake was reported to produce greater BP decreases in African-Americans [16,17] and a national strategy to reduce population levels of salt intake was finally introduced in the United Kingdom (UK) in 2003 [18,19].

Regarding the SA population, a systematic review[20] showed that BP levels are lower in Bangladeshis followed by Pakistanis and Indians, than in EU. The close relationship between diabetes and obesity has more recently driven the attention also to body weight reduction [21]. However, the potential contribution of other associated risk factors and behaviours was not previously investigated

The goal of this study was to perform a systematic review and meta-analysis of studies comparing BP levels of EU with those of SSA and SA with the aims of 1) estimating the absolute BP differences among panethnic subgroups, 2) assessing whether such differences are affected by the year of the study; and finally 3) assessing the potential contribution of other associated risk factors and behaviours. The term panethnicity usually refers to a collectivity consisting of distinct ethnic groups and cultures that experience themselves as having something in common [22] and was adopted to stress the limitations of grouping together, and collective labelling SSA and SA subjects of various separate ethnicities.

## Materials and Methods

### Literature review and data sources

Searches of MEDLINE, PubMed, EMBASE, Web of Science, and Scopus databases, from their inception to January 31st, 2015 without language restriction, were undertaken. The search terms ‘Asians’, ‘South Asians’, ‘Caribbean and Asian’, ‘Indians’, ‘East Indian’, ‘Pakistanis’, ‘Bangladeshis’, ‘Africans’, ‘African Caribbean’, ‘West Africans’, ‘Black’, ‘racial stock’, and ‘ethnic minority groups’ were combined with blood pressure, hypertension and Europe. Additionally, we hand-searched reference lists, screened citations of articles of interest and approached an international panel of experts in the field to identify additional published studies. Studies that were not published as full reports, such as conference abstracts and letters to the editor, were excluded.

### Inclusion criteria

There were no language restrictions. Studies were eligible for inclusion if they 1) compared BP levels in subjects originating from SSA or SA countries and living in Europe with those of EU; 2) reported office systolic(SBP) and diastolic BP(DBP) values with standard deviation, standard error, or 95% confidence intervals(CI); 3) enrolled adult individuals (age ≥16 years) at random from the general population.

### Data abstraction and assessment of study quality

Data were extracted from each paper with customised data extraction forms. Attempts were made to retrieve missing data after contacting the corresponding author. Missing variance measures were imputed using published formulas [23]. Three reviewers independently assessed the risk of bias of each of the included studies and discussed their assessments to achieve consensus. A score for quality, modified from the Newcastle-Ottawa scale, was used to assess appropriateness of research design, recruitment strategy, response rate, representativeness of sample, objectivity/reliability of outcome determination, power calculation provided, and appropriate statistical analyses. Score disagreements were resolved by consensus and a final agreed-upon rating was assigned to each study. We used the PRISMA (Preferred Reporting Items for Systematic reviews and Meta-Analyses) statement for reporting systematic reviews and meta-analyses as a guide for this study (S1 Text) [24], including the preparation of a protocol and analysis plan (S2 Text).

### Statistical analysis

Descriptive data are reported as means with 95% CI or median and range. Main outcome measures were the mean differences in SBP and DBP between SSA, SA, and EU. Data were pooled using a random-effect model and the I^2^ statistics was used to assess heterogeneity, where I^2^>50% was considered as evidence of significant heterogeneity [25]. In addition, we used Galbraith’s radial plots to visualize heterogeneity and the contribution of each study to the overall estimate[26]. Pre-specified subgroups included panethnic groups (SSA, SA, and EU) and gender. A generalised Q-statistics was used to evaluate differences among subgroups [27]. The potential unit-of-analysis issue related to multiple comparisons from a single study population (e.g. multi-ethnic studies where the EU control group was used more than once) was addressed by splitting the variance of the EU controls across the available comparisons [23].

Sources of heterogeneity were further investigated by random effect meta-regression analyses [25]. Potential effect modifiers, besides panethnicity and gender, included the study year and the differences in diabetes prevalence, age, body mass index(BMI), and smoking prevalence. Differences were calculated by subtracting the reported values of minority groups from those of EU populations. Studies including SA subjects were also categorized according to the country of origin and the main religion in the country of origin (Islam for Pakistan and Bangladesh; other for India, Sri Lanka, and Suriname)[28].

Model fit was assessed using the proportion of the between-study variance explained by the covariates (adjusted R^2^), along with a significance test for each covariate. The proportion of between-study variance explained by the covariates was calculated by comparing the residual between-study variance in the final model(*τ*^2^) with its value in a model without covariates ($\tau_{0}^{2}$). The adjusted R^2^, the relative reduction in the between-study variance, was calculated according to the formula:$\;R_{adj}^{2}\;=\;\frac{\left(\tau_{0}^{2}-\tau^{2}\right)}{\tau_{0}^{2}}$ [29]. To control for false-positive findings (type I error), when performing meta-regression with multiple covariates, we used the model F value and its statistical significance to assess whether there was evidence for an association of any of the covariates with the outcome, and a test based on random permutations to calculate multiplicity adjusted P value [29,30]. Publication bias was assessed by visual inspection of the funnel and regression test. To assess the impact of potentially “missing” studies on pooled estimates, sensitivity analyses were conducted using the Duval and Tweedie nonparametric “trim and fill” method using both the L_0_ and R_0_ estimator [31,32].

Two-sided tests for overall effects were considered significant at p≤0.05. Statistical analyses were performed using STATA 13 (StataCorp LP,College Station,Texas,USA) and R(R Foundation for Statistical Computing,Vienna,Austria).

## Results

### Characteristics of the included studies

**Fig 1:**
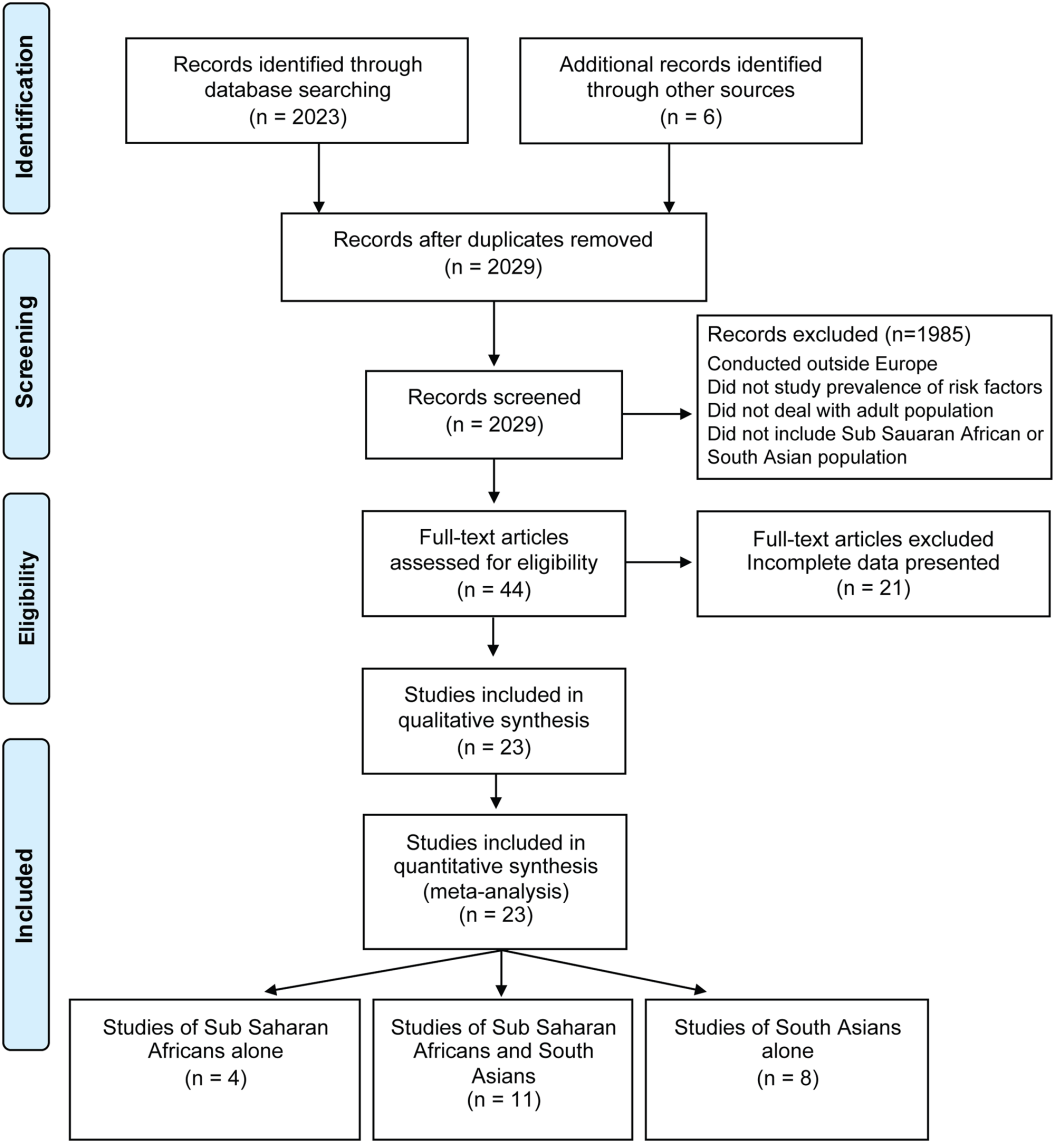
Flow chart of the selection process. Flow chart of the selection process according to the PRISMA Statement [24].

A total of 23 studies met inclusion criteria and were included in our meta-analysis (Fig 1).

The main characteristics of included studies are reported in Table 1. Data on SSA were provided in fifteen studies [33–47] (Table 2), nineteen studies providing data on SA [34,36,37,39,41,44–57](Table 3). Some of these studies involved several groups of individuals, so that a total of 17 and 26 comparison groups were available for analysis for SSA and SA respectively. Sample size ranged from 115 to 59,952 participants (median, 1,578), with a total of 9,070 SSA (3,894 men and 5,176 women), 18,421 SA(10,021 men and 8,400 women), and 130,380 EU (67,768 men and 65,612 women). The percentage of men ranged from 32% to 100% for SSA (median, 44%), from 41% to 100% for SA (median, 51%), and from 44% to 100% for EU (median, 51%).

**Table 1:** Characteristics of studies included in analysis.

Author [Ref]	Study Design	Time of study	Sampling frame	Country	Study populations (n =)	Indicator of panethnicity	Age range (years)	Method of BP measurement (device)	RR (%)	Gender	Quality score
Meade et al.[39]	PS	1977	FB	UK	EU(412), SSA(141)	CB	18–49	Not given	NR	M/W	7
Sever et al.[40]	CSS	1978	FB	UK	EU(62), SSA(53)	NR	24–58	A(Bosomat)	55	M/W	7
Haines et al.[30]	CSS	1983–1986	GPL	UK	EU(936), SSA(415)	CB	17–70	RZ	61	M/W	10
McKeigue et al.[46]	CSS	1985–1986	GPL	UK	EU(132), SA(121)	SR	35–69	RZ	81	M/W	10
Cruickshank et al.[31]	CSS	NR	GPL	UK	EU(101),SSA(106),SA(107)	SR	45–74	RZ	77	M/W	8
McKeigue et al.[36]	CSS	1988–1990	FB, GPL	UK	EU(1761),SSA(209),SA(1712)	N,CB	45–74	RZ	66 EU; 62 SA; 66 SSA	M/W	10
Knight et al.[47]	CSS	1989	FB	UK	EU(156), SA(96)	N,SR	20–65	A(Copal)	71 EU; 83 SSA	M	10
Chaturvedi et al.[32]	CSS	NR	GPL	UK	EU(585), SSA(581)	SR	40–69	RZ	58	M/W	10
Knight et al.[48]	CSS	NR	FB	UK	EU(160), SA(128)	N,SR	20–65	A(Copal)	71 EU; 83 SSA	M	10
Simmons et al.[52]	CSS	1986–1989	PL	UK	EU(5508), SA(4395)	CB	>20	RZ	NR	M/W	10
Cappuccio et al.[33]	CSS	1994–1996	GPL	UK	EU(524),SSA(549),SA(505)	SR	40–59	A(Arteriosonde)	64	M/W	10
Bhopal et al.[49]	CSS	1993/94 EU; 1995/97 SA	GPL	UK	EU(825), SA(684)	CB	25–74	Manual	64 EU; 67 SA	M/W	10
HSE 1999 [43]	CSS	1999	-	UK	EU(11884),SSA(719),SA(1973)	CB	>16	-	NR	M/W	10
Whitty et al.[41]	CS	1985–1988	OS	UK	EU(8973), SSA(360), SA(577)	SR	35–56	RZ	73	M/W	10
Lane et al.[34]	CS	1979/86 and 1996/97	FB	UK	EU(2067), SSA(394), SA(226)	SR	30–60	RZ	NR	M/W	9
HSE 2004. [47]	CSS	2004	-	UK	EU(9183), SSA(675), SA(1197)	CB	>16	-	NR	M/W	10
Agyemang et al.[42]	CSS	2001–2003	PL	NL	EU(508), SSA(581), SA(294)	SR	35–60	A(Omron M-4)	61	M/W	10
Lyratzopoulos et al.[50]	CSS	1989–1999	PL	UK	EU(9995), SA(9990)	N	35–60	Manual	NR	M/W	9
Glenday et al.[53,54]	CSS	2002	PL	NO	EU(11027), SA(1976)	CB	31–60	A(DINAMAP)	40	M/W	10
Gualdi-Russo et al.[38]	CSS	2000–2002	IC	I	EU(104), SSA(44), SA(78)	CB	17–65	Manual	67	M	7
Gray et al.[51]	CSS	2004–2007	GPL	UK	EU(4688), SA(1353)	SR	40–75	A(Omron)	22	M/W	9
Rabanal et al. [58]	CSS	1994–2003	PL	NO	EU(58698), SSA(183), SA(1071)	CB	40–65	-	58	M/W	10
Agyemang et al. [59]	PS	2011	PL	NL	EU(2097), SSA(4060), SA(2278)	CB	18–70	A(Microlife)	63	M/W	10

BP = Blood Pressure; RR = Response Rate; PS = prospective study; CSS = Cross Sectional Survey; CS = Cohort Study; FB = Factory based; GPL = General practices list; HC = Health centre; PL = Population List; OS = Office staff; IC = Immigrant centres; UK = United Kingdom; NL = The Netherlands; NO = Norway; I = Italy; EU = Europeans; SSA = Sub-Saharan Africans (including populations also defined as Africans, Afro-Caribbeans, and Black Caribbeans); SA = South Asians; CB = Country of birth; NR = Not Reported; N = Name; SR = Self-reported; RZ = random zero manometer; A = automated sphygmomanometer; M = men; W = Women.

**Table 2:** Age, mean prevalence of diabetes mellitus, body mass index, and smoking habit by gender among Sub-Saharan Africans and native Europeans.

Author [Ref]	Age (range)	MEN								WOMEN							
		Age (mean)		Diabetes (%)		BMI (kg/m^2^)		Smokers (%)		Age (mean)		Diabetes (%)		BMI (kg/m^2^)		Smokers (%)	
		SSA	EU	SSA	EU	SSA	EU	SSA	EU	SSA	EU	SSA	EU	SSA	EU	SSA	EU
Meade et al.[42]	18–49	35.5	34.7	-	-	24.5	24.4	47.9	47.9	38.6	36.9	-	-	27.4	23.6	7.3	59.0
Sever et al.[43]	24–58	42.0	42.0	-	-	-	-	-	-	44.0	43.0	-	-	-	-	-	
Haines et al.[33]	17–70	41.4	42.4	-	-	25.0	25.3	41.4	46.3	36.5	40.9			25.8	25.2	27.5	45.6
Cruickshank et al.[34]	45–74	57.0	62.2	41.0*	4.0*	26.0	26.2	-	-	56.6	60.3	13.0*	2.0*	29.1	26.3	-	
McKeigue et al.[39]	40–69	-	-	14.6*	4.8*	26.3	25.9	-	-	-	-	-	-	-	-	-	
Chaturvedi et al.[35]	40–64	55.0	55.0	12.9*	6.5*	26.0	26.4	30.0	37.0	55.0	55.0	17.7*	4.0*	29.1	26.0	8.0	35.0
Cappuccio et al.[36]	40–59	51.1	49.8	18.0*	7.0*	26.4	25.8	18.6	39.6	51.1	49.8	15.0*	5.0*	29.3	26.1	9.2	33.3
Whitty et al.[44]	35–56	45.8	43.8	5.0*	2.0*	-	-	17.0	15.0	45.8	43.8	5.0*	2.0*	-	-	9.0	26.0
HSE 1999 [46]	>16	43.1	45.0	7.8	3.3	27.1	26.6	35.0	27.0	41.3	45.4	7.9	2.5	28.0	26.4	25.0	27.0
Lane et al.[37]	>30	44.6	44.1	-	-	26.2	25.9	50.0	40.6	45.0	43.9	-	-	28.7	25.6	10.9	49.2
HSE 2004 (BC) [47]	>16	45.2	47.4	10.0**	4.3**	27.1	27.1	25.0	24.0	43.3	47.2	8.4**	3.4**	28.0	26.8	24.0	23.0
HSE 2004 (BA) [47]	>16	45.2	47.4	5.0**	4.3**	26.4	27.1	21.0	24.0	36.4	47.2	2.1**	3.4**	28.8	26.8	10.0	23.0
Agyemang et al.[45]	35–60	44.1	48.1	11.9	7.8	26.4	26.2	55.5	54.0	43.4	47.4	12.8	5.8	29.4	26.1	-	-
Gualdi-Russo et al.[41]	17–65	34.7	37.0	-	-	22.9	25.2	-	-	-	-	-	-	-	-	-	-
Rabanal et al.[58]	40–65	42.5	48.6	5.0	3.0	25.7	26.9	32.0	39.0	46.7	48.7	5.0	2.0	28.6	25.9	32.0	45.0
Agyemang et al.(AS)[59]	18–70	47.6	47.3	-	-	26.4	25.2	42.6	27.0	47.5	45.5	-	-	28.8	24.3	23.9	24.2
Agyemang et al.(G)[59]	18–70	47.1	47.3	-	-	26.8	25.2	7.8	27.0	43.9	45.5	-	-	29.5	24.3	2.4	24.2

BMI = body mass index; SSA = Sub-Saharan Africans; EU = Europeans; BC = Black Caribbeans; BA = Black Africans; AS = African Surinamese; G = G = Ghanians.

* = Oral Glucose Tolerance Test (75 g);

** = Self-reported doctor-diagnosed diabetes.

**Table 3:** Age, mean prevalence of diabetes mellitus, body mass index, and smoking habit by gender among South Asians and native Europeans.

Author [Ref]	Age(range)	MEN								WOMEN							
		Age (mean)		Diabetes (%)		BMI (kg/m^2^)		Smokers (%)		Age (mean)		Diabetes (%)		BMI (kg/m^2^)		Smokers (%)	
		SA	EU	SA	EU	SA	EU	SA	EU	SA	EU	SA	EU	SA	EU	SA	EU
McKeigue et al.[46]	35–69	50.7	50.9	22.0*	10.0*	23.9	26.6	82.0	45.0	49.7	51.4	23.0*	4.0*	23.7	26.1	22.0	39.0
Cruickshank et al.[31]	45–74	62.2	62.2	31.0*	4.0*	25.2	26.2	-	-	60.0	60.3	28.0*	2.0*	26.8	26.3	-	-
McKeigue et al.[36]	40–69	-	-	19.6*	4.8*	25.7	25.9	-	-	-	-	16.1*	2.3*	27.0	25.2	-	-
Knight et al.[47]	20–65	39.3	41.0	12.7*	4.5*	23.8	25.2	43.8	43.9	-	-	-	-	-	-	-	-
Knight et al.[48]	20–65	41.0	41.0	10.9*	4.4*	24.5	25.0	39.1	43.8	-	-	-	-	-	-	-	-
Simmons et al.[52]	20–60	-	-	12.4*	3.2*	-	-	24.0	48.0	-	-	11.2*	4.7*	-	-	3.0	47.0
Cappuccio et al.[33]	40–59	49.4	49.8	25.0*	7.0*	24.8	25.8	25.2	39.6	49.4	49.8	20.0*	5.0*	27.1	26.1	2.9	33.3
Bhopal et al.(Indians) [49]	25–74	50.7	54.2	16.0*	16.0*	26.9	26.1	14.0	32.0	52.4	54.0	20.0*	15.0*	27.9	26.0	1.0	31.0
Bhopal et al.(Pakistanis) [49]	25–74	52.2	54.2	35.0*	16.0*	26.6	26.1	32.0	32.0	48.3	54.0	34.0*	15.0*	27.8	27.0	5.0	31.0
Bhopal et al.(Bangladeshi)[49]	25–74	47.7	54.2	17.0*	16.0*	25.4	26.1	57.0	32.0	48.1	54.0	13.0*	15.0*	26.3	28.0	2.0	31.0
HSE 1999(Indians)[43]	>16	41.2	45.0	7.7**	3.3**	25.2	26.6	23.0	27.0	40.3	45.4	4.7**	2.5**	25.9	26.4	6.0	27.0
HSE 1999(Pakistanis)[43]	>16	37.6	45.0	8.7**	3.3**	25.4	26.6	26.0	27.0	34.6	45.4	5.3**	2.5**	26.5	26.4	5.0	27.0
HSE 1999(Bangladeshi)[43]	>16	39.2	45.0	10.6**	3.3**	23.8	26.6	44.0	27.0	33.9	45.4	5.9**	2.5**	24.1	26.4	1.0	27.0
Whitty et al.[41]	35–56	46.3	43.8	7.0*	2.0*	-	-	25.0	15.0	46.3	43.8	7.0*	2.0*	-	-	6.0	26.0
Lane et al. [34]	>30	44.5	44.1	-	-	25.2	25.9	31.3	40.6	-	-	-	-	-	-	-	-
HSE 2004 (Indians) [44]	>16	43.5	47.4	10.1**	4.3**	25.8	27.1	20.0	24.0	41.9	47.2	5.9**	3.4**	26.2	26.8	5.0	23.0
HSE 2004 (Pakistanis) [44]	>16	37.9	47.4	7.3**	4.3**	25.9	27.1	29.0	24.0	35.0	47.2	8.6**	3.4**	27.1	26.8	5.0	23.0
HSE 2004 (Bangladeshi) [44]	>16	38.8	47.4	8.2**	4.3**	23.8	27.1	40.0	24.0	35.0	47.2	5.2**	3.4**	25.7	26.8	2.0	23.0
Agyemang et al.[42]	35–60	44.3	48.1	24.8	7.8	26.4	26.2	48.5	54.0	45.0	47.4	25.8	5.8	27.5	26.1	-	-
Lyratzopoulos et al.[50]	35–60	44.2	45.6	-	-	25.9	26.7	27.4	43.6	43.7	45.7	-	-	26.4	26.0	7.0	34.4
Glenday et al.(Pakistanis)[53,54]	31–60	45.3	44.6	11.0*	2.0*	27.4	26.3	37.0	27.0	43.9	44.3	14.0*	1.0*	29.3	24.8	5.0	27.0
Glenday et al.(Sri Lankans)[53,54]	31–60	40.1	44.6	8.0*	2.0*	25.7	26.3	19.0	27.0	39.5	44.3	10.0*	1.0*	26.8	24.8	19.0	27.0
Gualdi-Russo et al.[38]	17–65	32.6	37.0	-	-	22.8	25.2	-	-	-	-	-	-	-	-	-	-
Gray et al.[51]	40–75	53.9	58.7	6.1*	3.4*	26.6	28.2	-	-	52.2	58.5	4.1*	2.2*	28.4	28.4	-	-
Rabanal et al.[58]	40–65	46.7	48.6	14.0	3.0	26.8	26.9	28.0	39.0	46.7	48.4	15.0	2.0	29.0	25.9	2.0	45.0
Agyemang et al.[59]	18–70	45.2	47.3	-	-	25.7	25.2	39.5	27.0	46.7	45.5	-	-	26.9	24.3	18.6	24.2

BMI = body mass index; SA = South Asians; EU = Europeans.

* = Oral Glucose Tolerance Test (75 g);

** = Self-reported doctor-diagnosed diabetes.

### Panethnicity and blood pressure

**Fig 2:**
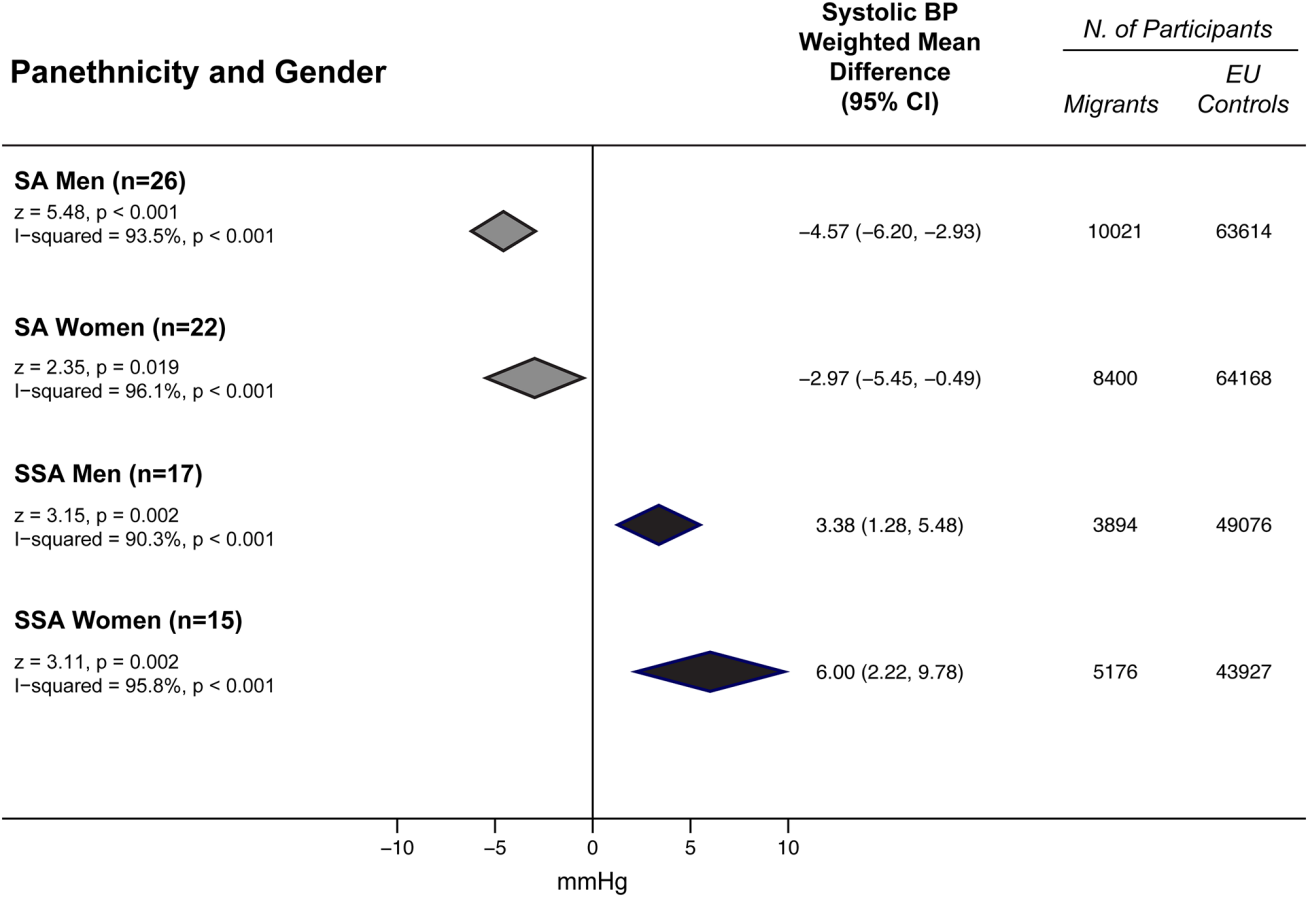
SBP differences between minority groups and EU participants by panethnicity and gender. Subgroup comparisons of the weighted mean difference of systolic blood pressure (BP) between minority groups and EU participants by panethnicity and gender. Diamonds denote the pooled estimates and 95% confidence intervals. SSA = Sub-Saharan Africans; SA = South Asians; EU = Europeans, “n” is the number of comparisons available for each subgroup.

**Fig 3:**
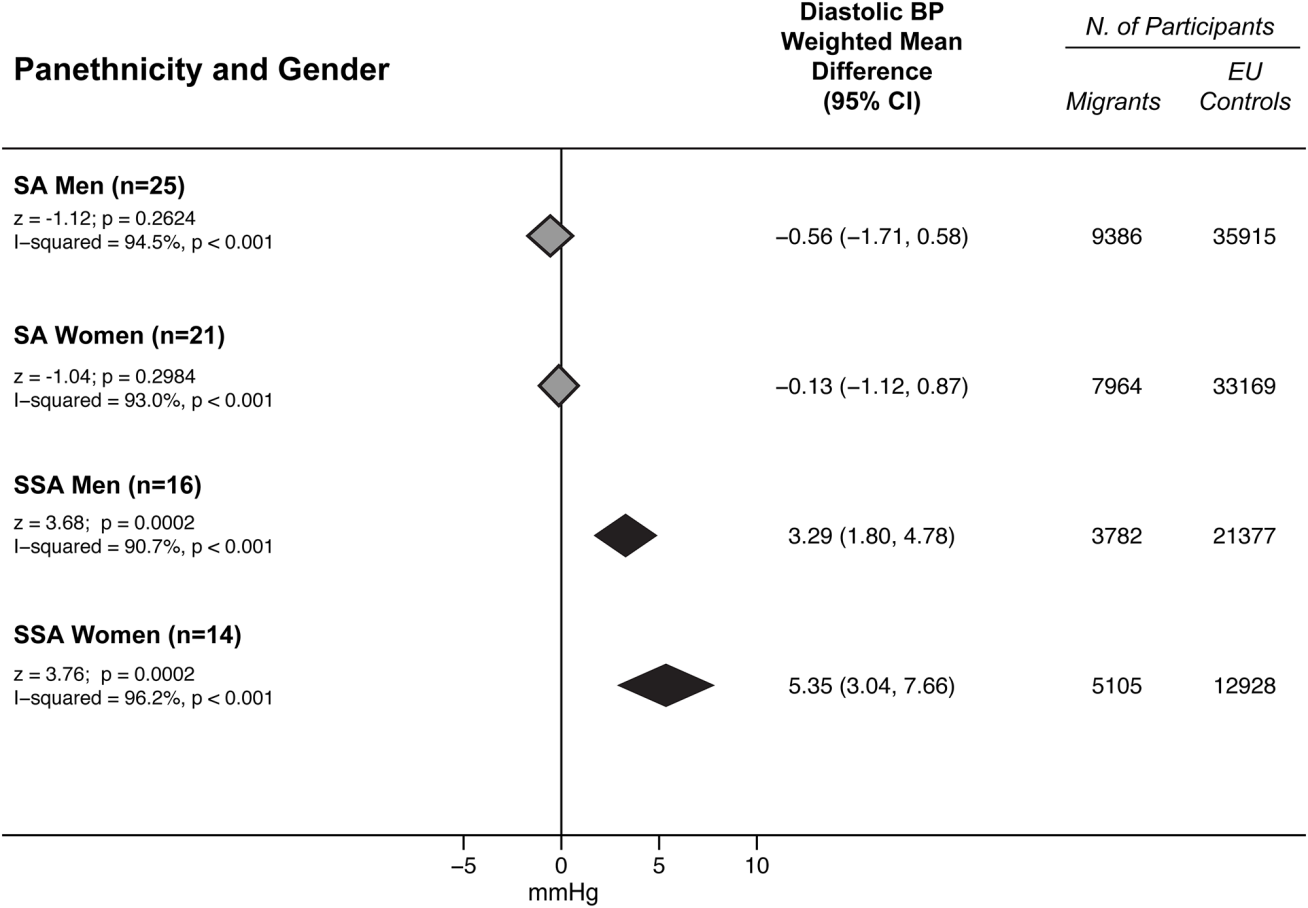
DBP differences between minority groups and EU participants by panethnicity and gender. Subgroup comparisons of the weighted mean difference of diastolic blood pressure (BP) between minority groups and EU participants by panethnicity and gender. Diamonds denote the pooled estimates and 95% confidence intervals. SSA = Sub-Saharan Africans; SA = South Asians; EU = Europeans, “n” is the number of comparisons available for each subgroup.

SSA had higher BP values than EU subjects for both SBP (3.38 mmHg; 95% CI 1.28 to 5.48 in men; 6.00 mmHg, 95% CI 2.22 to 9.78 mmHg in women) (Fig 2) and DBP (3.29 mmHg, 95% CI 1.80 to 4.78 mmHg in men; 5.35 mmHg, 95% CI 3.04 to 7.66 in women) (Fig 3).

On the contrary, SA had SBP values lower than EU (-4.57 mmHg, 95% CI -6.20 to -2.93 mmHg in men; -2.97 mmHg, 95% CI -5.45 to -0.49 mmHg in women) (Fig 2). They also tended to have lower, albeit not significantly, DBP values (-0.56 mmHg, 95% CI -1.71 to 0.58 in men; -0.13 mmHg, 95% CI -1.12 to 0.87 in women) (Fig 3). Effect sizes of individual studies on South Asians are reported in S1 Fig (for systolic BP) and in S2 Fig (for diastolic BP). Effect sizes of individual studies on Sub-Saharan Africans are reported in S3 Fig (for systolic BP) and in S4 Fig (for diastolic BP).

In these analyses, however, we found evidence of statistical heterogeneity among studies. The I-squared statistic ranged between 90.3% and 96.1% for SBP, and between 90.7% and 96.2% for DBP. Notably, gender did not contribute to heterogeneity within subgroups. A sensitivity analysis, excluding one study at the time, neither reduced heterogeneity nor affected the overall estimates to a noticeable extent (S1 and S2 Tables). Visual inspection of the funnel plot (S5 Fig), and regression tests of funnel plot asymmetry (p = 0.31 and p = 0.72 for SBP and DBP, respectively) showed no formal evidence of publication bias. Application of trim and fill to the SBP and DBP meta-analysis did not reveal missing studies.

Visual inspection of the funnel plot (S5 Fig), and regression tests of funnel plot asymmetry (p = 0.31 and p = 0.72 for SBP and DBP, respectively) showed no formal evidence of publication bias. Application of trim and fill to the SBP and DBP meta-analysis did not reveal missing studies.

**Fig 4:**
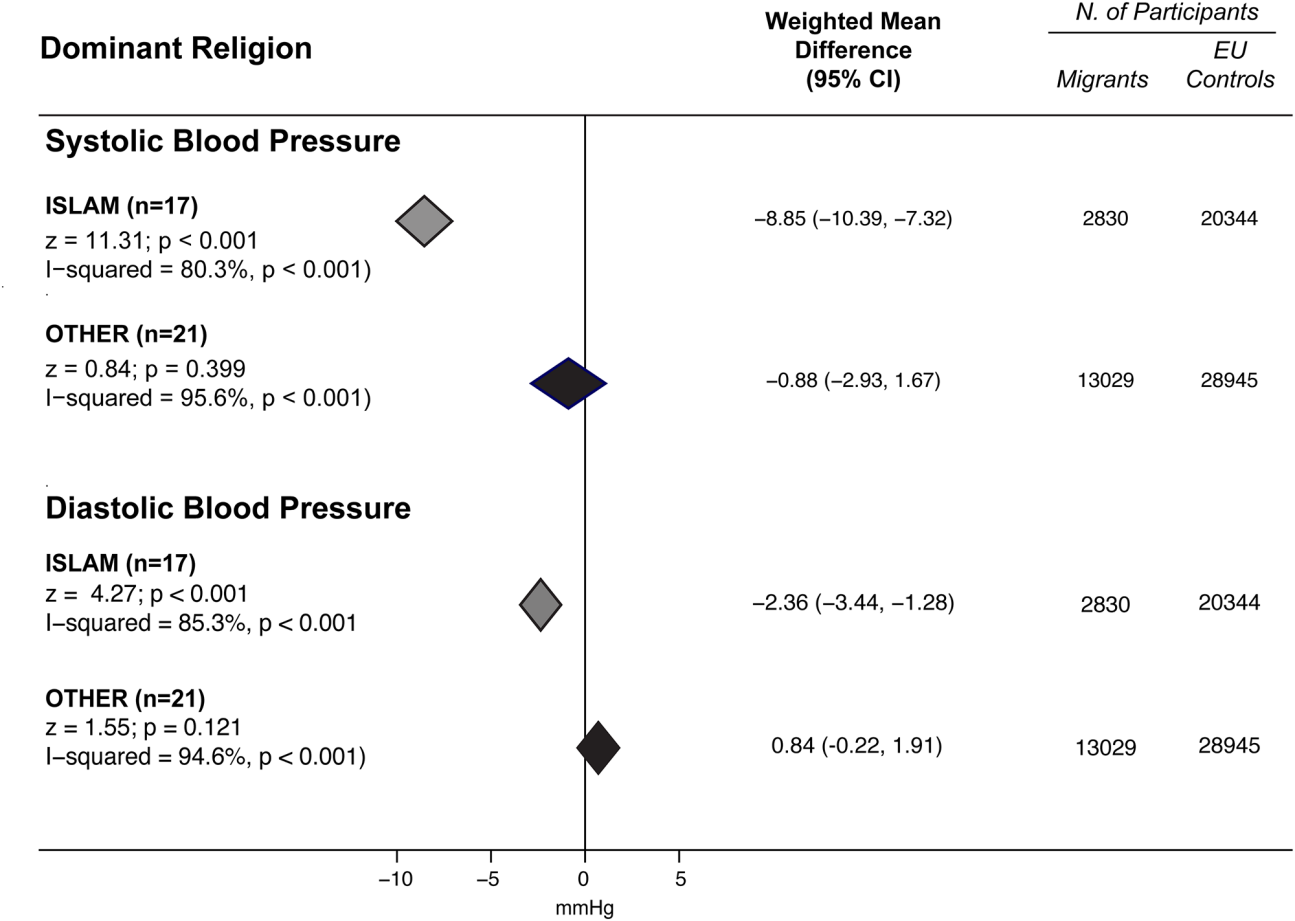
BP differences between subjects originating from SA countries and EU participants by dominant religion in the country of origin. Subgroup comparisons of the weighted mean difference of systolic and diastolic blood pressure between South Asians (SA) and European (EU) participants by dominant religion in the country of origin. The term "others" refers to comparisons performed between EU participants and subjects who originated from countries with a different dominant religion. Diamonds denote the pooled estimates and 95% confidence intervals, “n” is the number of comparisons available for each subgroup.

To further investigate sources of heterogeneity among studies on SA participants, studies were grouped according to the country of origin. The Q-statistic for subgroups differences was highly significant for SBP and DBP in both genders (p<0.001 for all). Interestingly, BP differences were larger in subjects coming from Bangladesh and Pakistan for both SBP (S6 Fig) and DBP (S7 Fig). When studies were grouped according to the dominant religion in the country of origin, participants from Muslim countries showed significantly lower BP values than EU for both SBP (-9.22 mmHg 95% CI -11.44 to -7.00 in men, and -8.47 mmHg, 95% CI -10.75 to -6.18 in women) and DBP (-3.23, 95% CI -5.24 to -1.23 in men, and -1.57, 95% CI -2.28 to -0.85 in women). Differences between Muslim and non-Muslim participants were significant (Fig 4) for both SBP and DBP (p<0.001 for all comparisons) regardless of gender (interaction p = 0.644 and p = 0.126 for SBP and DBP respectively).

### Role of potential effect modifiers

Because heterogeneity among studies and subgroups was substantial (I-squared > 85%), we used multivariate random-effect meta-regression to investigate the role of potential effect modifiers of BP differences (panethnicity, gender, study year, and the differences in diabetes prevalence, BMI, age at examination, and smoking prevalence between minority groups and EU). Panethnicity had a highly significant effect on both SBP (Table 4) and DBP mean differences (Table 5). In both models, gender and the interaction terms were not significant. We also tested study quality as a potential effect modifier in a meta-regression model (S3 Table) and found no evidence of a significant effect for both SBP and DBP.

**Table 4:** Random effect meta-regression estimates of systolic blood pressure for panethnicity, gender and their interaction.

Variables	Point Estimate	95% CIs	p-value	Multiplicity adjusted p-value
**Panethnicity** (SSA vs. SA)	8.014	4.369 to 11.660	<0.001	<0.001
**Gender** (Women vs. Men)	1.618	-1.762 to 4.998	0.348	0.650
***Interaction Term***				
Panethnicity x Gender	0.860	-4.529 to 6.249	0.755	0.980

SSA = Sub-Saharan Africans; SA = South Asians; EU = Europeans. Systolic blood pressure Model (number of comparisons = 80): Intercept: -4.5857 mmHg (-6.862 to -2.310; p<0.001); Proportion of between-study variance explained (Adjusted R-squared) = 34.45%; Joint test for all covariates F = 13.34, p<0.0001

**Table 5:** Random effect meta-regression estimates of diastolic blood pressure for panethnicity, gender and their interaction.

Variables	Point Estimate	95% CIs	p-value	Multiplicity adjusted p-value
**Panethnicity** (SSA vs. SA)	3.896	1.757 to 6.035	<0.001	<0.001
**Gender** (Women vs. Men)	0.366	-1.587 to 2.318	0.713	0.980
***Interaction Term***				
Panethnicity x Gender	1.593	-1.569 to 4.755	0.323	0.700

SSA = Sub-Saharan Africans; SA = South Asians; EU = Europeans. Diastolic blood pressure Model (number of comparisons = 76): Intercept: -0.598 mmHg (-1.912 to 0.716); p = 0.373); Proportion of between-study variance explained (Adjusted R-squared) = 33.02%; Joint test for all covariates F = 11.91, p<0.0001

In further multivariate meta-regression analyses (including all candidate effect modifiers), the weighted mean SBP difference between minority groups and EU, was largely influenced by panethnicity and by the difference in diabetes prevalence (Table 6) while study year, difference in BMI, age at examination, and the difference in smoking prevalence did not reach formal statistical significance. The proportion of residual between-study variance (final model τ^2^ = 8.37 and model without covariates τ^2^ = 39.35) explained by including the above covariates in the model was large (Adjusted R-squared 78.73%), but still significantly different from zero(likelihood ratio test of τ^2^ = 0, p<0.001).

**Table 6:** Weighted mean difference in systolic blood pressure in relation to explanatory variables in multivariate meta-regression model including all potential effect modifiers.

Variables	Point Estimate	95% CIs	p-value	Multiplicity Adjusted p-value
**Panethnicity** (SSA vs. SA)	7.674	4.757 to 10.591	<0.001	<0.001
**Diabetes Prevalence** (1% difference)	0.315	0.129 to 0.501	0.001	0.004
**Study Year** (1 year)	0.114	-0.189 to 0.417	0.460	0.990
**Body Mass Index** (1 kg/m^2^ difference)	0.165	-0.868 to 1.198	0.755	1.000
**Age at examination** (1 year difference)	0.547	0.058 to 1.037	0.028	0.120
**Gender** (Women vs. Men)	-3.101	-6.699 to 0.496	0.091	0.380
**Smoking Prevalence** (1% difference)	-0.194	-0.397 to 0.009	0.062	0.280

Differences were calculated by subtracting the average values of Minority groups from those of Europeans. SSA = Sub-Saharan Africans; SA = South Asians; EU = Europeans. Model for Systolic blood pressure (number of comparisons = 42): Proportion of between-study variance explained (Adjusted R-squared) = 78.73%; joint test for all covariates F = 14.33, p<0.0001

**Fig 5:**
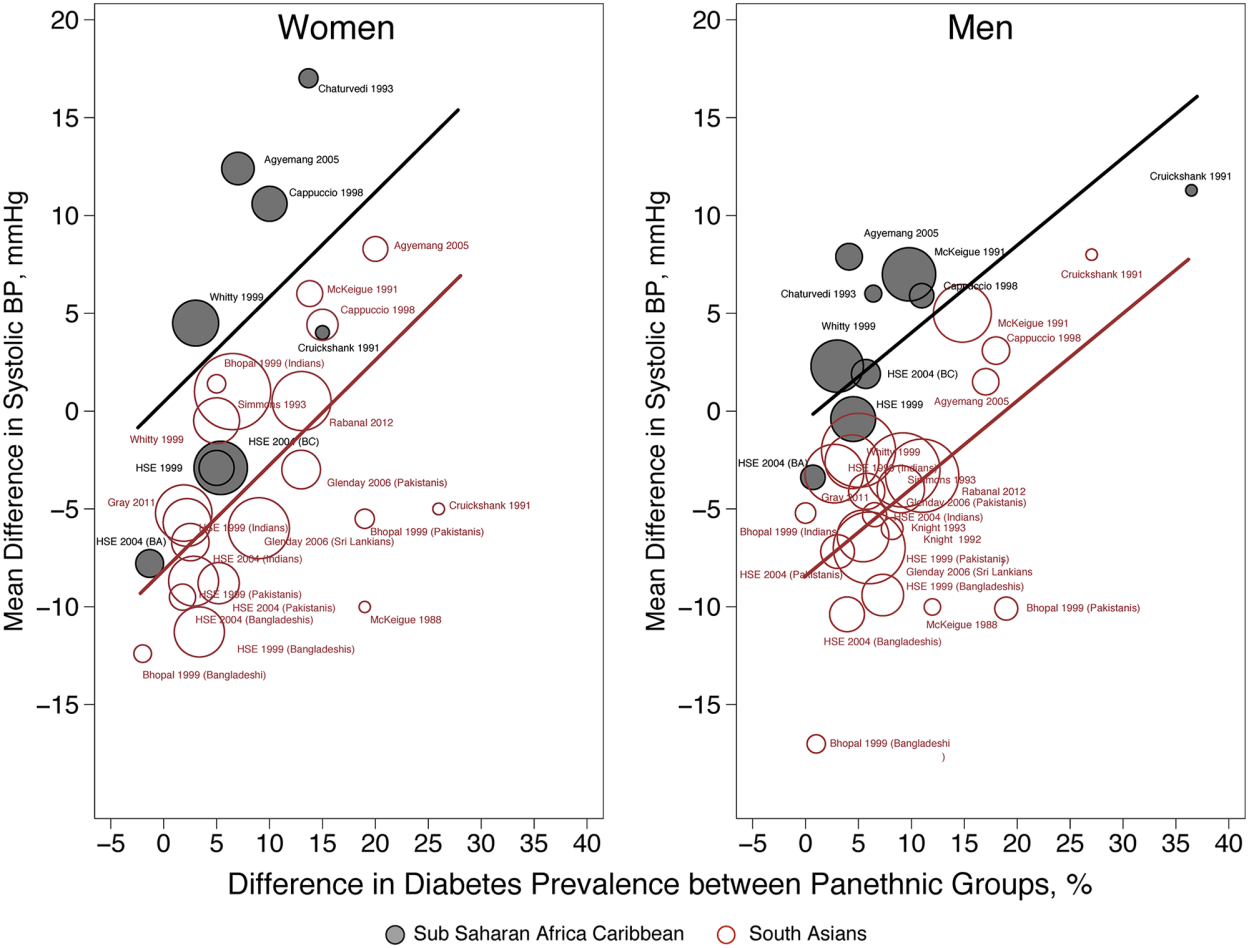
Gender specific meta-regression model. **Women** (number of comparisons = 28). Intercept: -8.260 mmHg (-12.167 to -4.352; p<0.001); Ethnicity: 8.695 mmHg (3.751 to 13.640; p = 0.001); Difference in Diabetes Prevalence: 0.543 mmHg (0.207 to 0.880; p = 0.002). Proportion of between-study variance explained (Adjusted R-squared) = 45.81%; joint test for all covariates F = 9.79, p<0.0017. **Men** (number of comparisons = 31). Intercept: -7.730 mmHg (-10.268 to -5.193; p<0.001); Ethnicity: 8.496 mmHg (5.234 to 11.759); p<0.001); Difference in Diabetes Prevalence: 0.481 mmHg (0.162 to 0.600; p<0.001). Proportion of between-study variance explained (Adjusted R-squared) = 60.73%; joint test for all covariates F = 18.04, p<0.001.

Representative plots of the joint effect of panethnicity and difference in diabetes prevalence on the weighted mean SBP difference for women and men are shown in Fig 5.

For DBP the weighted mean difference was influenced only by panethnicity (4.24 mmHg, 95% CI 1.77 to 6.71, p = 0.001) whereas none of the other tested effect modifiers had a significant effect.

## Discussion

The results of this systematic review and meta-analysis show that: a) SSA living in Europe have higher BP levels than EU; in the highly heterogeneous group of SA, BP levels are on average lower than in EU, mainly owing to the finding of lower BP values in people coming from Muslim countries; b) in meta-regression analyses the weighted mean SBP difference between minority groups and EU, was not influenced by the year of the survey; c) the presence of diabetes, irrespective of origin, is associated with higher BP levels.

These results lead to three important considerations: a) prevention strategies implemented in Europe played a key role in reducing BP in general population and in reducing the incidence of CV disease in Europe; however, the difference of BP levels between SSA and EU was consistent over years thus suggesting the importance of population specific intervention strategies; b) the finding of lower BP levels in SA Muslim populations suggests the importance of as yet untapped lifestyle and behavioral habits of such populations (religion being a likely proxy for them) that may represent an advantage reflecting a lower predisposition towards the development of hypertension; c) the additive effect of diabetes, independent from origin, in explaining the findings of elevated BP levels emphasizes the need to develop new strategies for the prevention and control of hypertension and ensuing CV disease in groups at higher prevalence of diabetes.

### Sub Saharan Africans

SSA individuals living in Europe have higher BP values than EU independently of gender. Consistent with raised BP levels, an excess risk of stroke and renal disease was observed for SSA living in Europe [5,6]. The term SSA was adopted to accommodate the population divisions used in the papers analysed although it is to be acknowledged the heterogeneity of this SSA group in terms of language, diet, and religious practices [2,60]. Whilst there may be genetic markers of predisposition to hypertension in SSA populations [2,61,62] it is clear from international and migration studies that their higher burden of hypertension is highly modifiable with lifestyle changes and adaptations to host environments [2,63,64]. This consideration encouraged public and health care systems to intervene in this group. Awareness of the higher burden of hypertension in SSA subjects was reported to be high amongst European physicians who more correctly recognize and diagnose hypertension in SSA than in other ethnic groups [36]. Likewise specific prevention strategies for hypertension in SSA (mainly low sodium diet) exist [65]. In addition, minority SSA (or SA) groups at the end of the 1970s were likely to be different from their counterparts at the end of the 2000s in terms of immigration reasons, habits, and socioeconomic status. All these factors could eventually influence the risk of high BP. The studies included in this meta-analysis were carried out in Europe over a period of over 30 years (from 1977 to 2015), and may give us the opportunity to assess whether BP differences in the SSA group were influenced by time. However, when the potential interaction between time trends for differences in BP and other CV risk factors, body mass index and diabetes was taken into account, the heterogeneity of the studies was not influenced by the time of data collection. Therefore limitations of prevention strategies so far implemented in effectively reaching minority subgroups and in modifying their CV risk profile are evident.

### South Asians

BP levels are not significantly higher in SA than in EU. This finding apparently does not fit with the higher incidence of stroke and CV death in SA living in Europe as compared with EU [5,8,66,67]. The heterogeneity of studies carried out in SA is however large. Beside the high prevalence of diabetes mellitus, uniformly higher in SA than in many other populations, genetic predisposition may also contribute to the high CV disease burden in SA [68,69]. The so-called SA group is highly heterogeneous also because SA living in Europe cover a wide range of migrant profiles (professionals, business elites, unskilled labourers, refugees). In addition, SA ethnic roots originate from the Indian subcontinent, a large geographic area that includes India, Pakistan, Sri Lanka, Nepal, and Bangladesh, with important differences in diet, culture, and lifestyle among different populations and religions. BP variations between SA populations in Europe with different countries of origin have already been reported [52] and important differences between Hindus and Muslims have been found [63,70]. In the present analysis, when study populations are categorized according to the main religion of the country of origin, this factor also significantly contributes to the heterogeneity. The country of origin and religion are proxies for other risk factors and behaviours (i.e. alcohol use, vegetarian diet, use of different cooking procedures) [71] that may explain some of the differences. Unfortunately, data on alcohol consumption, and diet are not available in most of included cohorts. In addition, the classification of studies including mixed populations as 'other' [53], might lead to a misrepresentation. The use of the main religion in the country of origin as a proxy for religion of the migrant populations, might also lead to a biased sample, with the clearest example being minority populations fleeing persecution from majority populations. However, notwithstanding the presence of Muslim subjects among populations classified as 'other' and the presence of non-Muslim individuals within the Muslim group, differences are evident. It might thus be important to consider this aspect in culturally-specific strategies for prevention [72].

Lifestyle modification can also slow down the progression of diabetes mellitus. Although mean BMI seems comparable between SA and EU, it should be considered that compared with EU, SA subjects have increased abdominal visceral fat and greater insulin resistance at similar levels of BMI [73]. It has indeed been argued that at comparable values of BMI, SA subjects may have a higher risk of CV disease than EU [3,39,74,75]. Data on waist circumference are not available in most of included cohorts. In spite of this, and although the measurement of BMI might not be a key factor to explain the observed heterogeneity [76], it is essential to encourage the control of body weight in migrant populations because of its relationship with diabetes.

### Strengths and limitations

This meta-analysis has some strengths: the overall large size enabling subgroup analyses, and the common study strategies. The study includes cohorts from all over Europe, although the data are predominantly UK-based.

Because of the nature of the studies and the large statistical heterogeneity, this systematic review has some limitations. When working with observational studies there is a good chance that heterogeneity exists between studies and that heterogeneity can be more extreme in observational studies than interventional studies [77]. Indeed, large heterogeneity can be problematic if a single summary measure is used[78], therefore we stratified by study level features and used regression analysis to quantitatively assess the extent to which pooled estimates varied along with these features[77–79]. We believe that our careful and comprehensive approach reduces the impact of heterogeneity on estimates and can make an important contribution to debates on public health policy.

Despite adjustment for known potential confounders, we cannot rule out the possibility that the observed associations are confounded by other unmeasured factors such as dietary sodium and potassium. This problem is made more complex by scenarios that include possible differences in the use of antihypertensive treatment among subgroups. In the Health Survey for England (HSE) study [46], treatment rates were found to be highest among black men and women. Among those on antihypertensive medication, the odds of having BP controlled (SBP <160 mm Hg and DBP<95 mm Hg) did not differ among the three groups of older men but was reduced in older SA women, compared with white women.

Average BP values measured at the population level may be influenced by both the number of measurements performed at a single visit, and by the number of visits considered by the study protocol. Although these methodological aspects may sometimes limit the possibility to compare absolute results obtained in different studies, they unlikely influenced the interpretability of our results, the same methodology being adopted for the different ethnic groups within the same study. The same consideration is also valid for diabetes, the same diagnostic criteria being adopted for different groups within the same study.

Finally, the pragmatic approach we adopted to accommodate studied populations in two large panethnic groups, limits the possibility to investigate in details the role of ethnicity. The essence of ethnicity embodies shared origins or social background, culture, language and religious traditions that are distinctive, maintained between generations, which lead to a sense of identity, and group.

SSA and SA groups are highly heterogeneous so that future research should prioritize the examination of variation within the two groups.

## Conclusions

The control of hypertension among minority groups is crucial for addressing social inequalities in health care. To avoid escalation of such inequalities, two main strategies might be considered. From the point of health care delivery, easier access to services as well as availability and affordability of antihypertensive drugs should be facilitated and awareness of specific needs among practitioners should be improved. However these interventions might be still ineffective in transferring the concept of prevention to migrant and vulnerable populations. Public health preventive strategies should match health care provision. It has taken decades to educate and increase awareness amongst EU citizens on the importance of prevention but only few European Union member states so far offer all undocumented migrants the opportunity to have their BP treated [1,9,14]. Ignoring this when immigration is increasing might halt the virtuous reduction of CV events that has occurred in Europe over the last decades.

## Supporting Information

S1 Text Newcastle—Ottawa Quality Assessment Scale (adapted for cross sectional studies). (PDF)

S2 Text Systematic Review Protocol. (PDF)

S1 Table Influential analysis (Random effects model) for systolic blood pressure weighted mean difference (WMD). (PDF)

S2 Table Influential analysis (Random effects model) for Diastolic blood pressure weighted mean difference (WMD). (PDF)

S3 Table Effect of study quality on estimates of systolic (A) and diastolic (B) blood pressure (random effect metaregression). (PDF)

S1 Fig Effect sizes of individual studies on South Asians for systolic BP. Forest plot of Systolic Blood Pressure mean differences between South Asians and Europeans. Estimates (95% CIs) are denoted by black boxes (black lines). A red diamond represents the pooled estimates for men and women, where diamond width corresponds to 95% CI bounds, “n” is the number of comparisons available within each subgroup.
(PDF)

S2 Fig Effect sizes of individual studies on South Asians for diastolic BP. Forest plot of Diastolic Blood Pressure mean differences between South Asians and Europeans. Estimates (95% CIs) are denoted by black boxes (black lines). A red diamond represents the pooled estimates for men and women, where diamond width corresponds to 95% CI bounds, “n” is the number of comparisons available within each subgroup.
(PDF)

S3 Fig Effect sizes of individual studies on Sub-Saharan Africans for systolic BP. Forest plot of Systolic Blood Pressure mean differences between Sub Saharan Africans and Europeans. Estimates (95% CIs) are denoted by black boxes (black lines). A red diamond represents the pooled estimates for men and women, where diamond width corresponds to 95% CI bounds, “n” is the number of comparisons available within each subgroup.
(PDF)

S4 Fig Effect sizes of individual studies on Sub-Saharan Africans for diastolic BP. Forest plot of Diastolic Blood Pressure mean differences between Sub Saharan Africans and Europeans. Estimates (95% CIs) are denoted by black boxes (black lines). A red diamond represents the pooled estimates for men and women, where diamond width corresponds to 95% CI bounds, “n” is the number of comparisons available within each subgroup.
(PDF)

S5 Fig Funnel plots for publication bias. Funnel plots for publication bias. Each comparison is plotted by its effect size on the horizontal axis and its precision on the vertical axis.
(PDF)

S6 Fig Systolic BP differences between South Asians and Europeans by Country of origin. Subgroup comparisons of the weighted mean difference of systolic blood pressure between South Asians (SA) and European participants (EU) by country of origin. The term "others" refers to comparisons where the Country of origin of SA subjects was not specified. Diamonds denote the pooled estimates and 95% confidence intervals, “n” is the number of comparisons available within each subgroup.
(PDF)

S7 Fig Diastolic BP differences between South Asians and Europeans by Country of origin. Subgroup comparisons of the weighted mean difference of diastolic blood pressure between South Asians (SA) and European participants (EU) by country of origin. The term "others" refers to comparisons where the Country of origin of SA subjects was not specified. Diamonds denote the pooled estimates and 95% confidence intervals, “n” is the number of comparisons available within each subgroup.
(PDF)

S8 Fig Galbraith’s radial plots for Systolic and Diastolic BP. Precision is on the horizontal axis plotted against the individual standardized effect sizes (vᵢ is the sampling variance of the observed effect size (ES) and τ² is the amount of heterogeneity, see methods for details). The central solid line represents the overall effect. Potential outliers (open symbols) lie either above or below the upper and lower confidence limits (dotted lines).
(PDF)

## Abbreviations

SSA: Sub Saharan Africa
SA: South Asians
EU: Europeans
SBP: Systolic Blood Pressure
DBP: Diastolic Blood Pressure
BP: Blood Pressure
CV: Cardiovascular
UK: United Kingdom
BMI: Body Mass Index
HSE: Health Survey for England
RR: Response Rate
PS: Prospective Study
CSS: Cross Sectional Survey
CS: Cohort Study
FB: Factory Based
GPL: General Practices List
HC: Health Centre
PL: Population List
OS: Office staff
IC: Immigrant centres
NL: The Netherlands
NO: Norway
I: Italy
CB: Country of Birth
NR: Not Reported
N: Name
SR: Self-Reported
RZ: Random Zero manometer
A: Automated sphygmomanometer
M: Men
W: Women
