# Supplementary material for: Panethnic Differences in Blood Pressure in Europe: A Systematic Review and Meta-Analysis
Source: PLoS One. 2016 Jan 25;11(1):e0147601. doi: 10.1371/journal.pone.0147601 (PMC4725677; doi:10.1371/journal.pone.0147601)
Supplement: S1 Text — (PDF) [file pone.0147601.s001.pdf]

S1 Text
NEWCASTLE - OTTAWA QUALITY ASSESSMENT SCALE
(adapted for cross sectional studies)

Selection: (Maximum 5 stars)

1) Representativeness of the sample:
    a) Truly representative of the average in the target population. * (all subjects or random sampling)
    b) Somewhat representative of the average in the target population. * (non-random sampling)
    c) Selected group of users.
    d) No description of the sampling strategy.
2) Sample size:
    a) Justified and satisfactory. *
    b) Not justified.
3) Non-respondents:
    a) Comparability between respondents and non-respondents characteristics is established, and the response rate is satisfactory. *
    b) The response rate is unsatisfactory, or the comparability between respondents and non-respondents is unsatisfactory.
    c) No description of the response rate or the characteristics of the responders and the non-responders.
4) Ascertainment of the exposure (risk factor):
    a) Validated measurement tool. **
    b) Non-validated measurement tool, but the tool is available or described.*
    c) No description of the measurement tool.

Comparability: (Maximum 2 stars)

1) The subjects in different outcome groups are comparable, based on the study design or analysis. Confounding factors are controlled.
    a) The study controls for the most important factor (select one). *
    b) The study control for any additional factor. *

Outcome: (Maximum 3 stars)

1) Assessment of the outcome:
    a) Independent blind assessment. **
    b) Record linkage. **
    c) Self report. *
    d) No description.
2) Statistical test:
    a) The statistical test used to analyze the data is clearly described and appropriate, and the measurement of the association is presented, including confidence intervals and the probability level (p value). *
    b) The statistical test is not appropriate, not described or incomplete.

This scale has been adapted from the Newcastle-Ottawa Quality Assessment Scale for cohort studies to perform a quality assessment of cross-sectional studies for the systematic review, “Are Healthcare Workers’ Intentions to Vaccinate Related to their Knowledge, Beliefs and Attitudes? A Systematic Review”.

We have not selected one factor that is the most important for comparability, because the variables are not the same in each study. Thus, the principal factor should be identified for each study.

In our scale, we have specifically assigned one star for self-reported outcomes, because our study measures the intention to vaccinate. Two stars are given to the studies that assess the outcome with independent blind observers or with vaccination records, because these methods measure the practice of vaccination, which is the result of true intention.
