# Supplementary material for: Panethnic Differences in Blood Pressure in Europe: A Systematic Review and Meta-Analysis
Source: PLoS One. 2016 Jan 25;11(1):e0147601. doi: 10.1371/journal.pone.0147601 (PMC4725677; doi:10.1371/journal.pone.0147601)
Supplement: S2 Text — (PDF) [file pone.0147601.s002.pdf]

S2 Text. Systematic Review Protocol

Title:
PANETHNIC DIFFERENCES IN BLOOD PRESSURE IN EUROPE

Background & Review question(s)
The high rate of influx and settlement of migrant populations into Europe is changing the dynamics of regional population growth, and may pose important challenges for public health and clinical care. Migrants from Sub Saharan Africa (SSA) and South Asia (SA) living in Europe have higher risk of stroke than native Europeans (EU). Elevated blood pressure and cardio-metabolic risk factors are conditions that increase the risk of cardiovascular events such as stroke.
The goal of this study is to perform a systematic review and meta-analysis of observational studies comparing BP levels of EU with those of SSA and SA.
Aims
1) provide quantitative estimates of BP differences between migrants and native Europeans
2) assess the impact of demographic, geographical, and cultural factors on BP differences
3) assess the impact of associated cardio-metabolic risk factors and time trends on BP differences

Searches
A comprehensive literature search of the following databases will be conducted using research methodology filters:
1. MEDLINE
2. EMBASE
3. SCOPUS
4. Web of Science

Types of study to be included
Observational studies including:
1. Cross-sectional studies
2. Cohort studies

Condition or domain being studied
Elevated blood pressure is a condition that increases the risk of cardiovascular diseases such as stroke.

Participants & studies
Inclusion
Adult subjects originating from SSA or SA countries and living in Europe and native Europeans

Studies reporting office systolic (SBP) and diastolic (DBP) blood pressure values with standard deviation, standard error, or 95% confidence intervals (CI);

Exclusion
SSA or SA not living in Europe
Studies not reporting BP values

Exposure(s)
Blood pressure
Diabetes
Obesity
Smoking
Other CV risk factors
Socio-demographic characteristics

Context
European Countries

Exclusion
SSA and/or SA migrants living outside Europe

Outcome(s)
Primary outcome
Differences blood pressure - systolic /diastolic - by gender and panethnic group

Secondary outcomes
Temporal trends in BP differences
Relationship between demographic, geographical, and cultural factors on BP differences

Data extraction, (selection and coding)
Titles and abstracts of relevant studies will be retrieved using search strategy and methodology filters from the selected databases (PAM and GReb). Additional searches will be conducted independently by two authors (PAM and EP).
Review data will be extracted using standard protocol and data extraction form. Disagreement or any discrepancies will be resolved by consensus, and contact will also be made to the corresponding author for clarity and/or additional data.

Risk of bias (quality) assessment
The methodological quality of the studies to be included will be assessed using the Newcastle Ottawa quality score. Disagreements will be resolved by consensus

Strategy for data synthesis
All the included studies will be described and estimates of BP differences for the Panethnic groups will be calculated. These will be pooled where appropriate using

random effect meta-analysis. Heterogeneity will be assessed by I-squared statistics (I-squared >50% will be considered as evidence of significant heterogeneity) and publication bias will be assessed using funnel plot asymmetry and regression based tests.

Analysis of subgroups or subsets

Subgroup analyses will be performed to evaluate BP differences across demographic, geographical, and cultural lines. These include;

1. Gender (male/female)
2. Panethnic group (SSA and SA)
3. Country of origin
3. Dominant Religion in the country of origin (Islam vs. Non-Islam)

Meta-regression analysis

Sources of heterogeneity will be investigated by random effect meta-regression analyses. Anticipated potential effect modifiers, besides panethnicity and gender, to be considered will include: survey year, age, diabetes prevalence, body mass index (BMI), and smoking habits.

Dissemination plans

This review is planned to be presented at relevant conferences and published in peer-reviewed journal.

Contacts details for further information

Pietro Amedeo Modesti
Dept of Medicina Sperimentale e Clinica, University of Florence;
Largo Brambilla 3, 50134 Florence, ITALY.
pamodesti@unifi.it;

Organisational affiliation of the review

ESH Working Group on Cardiovascular Risk in Low Resource Settings
http://www.eshonline.org/Communities/WorkingGroups.aspx

Review team

Pietro Amedeo Modesti, University of Florence
Gianpaolo Reboldi, University of Perugia
Eleonora Perruolo, University of Florence

Collaborators

Francesco P. Cappuccio, University of Warwick
Charles Agyemang, University of Warwick
Giuseppe Remuzzi, Istituto di Ricerche Farmacologiche Mario Negri, Bergamo, Italy
Gianfranco Parati, University of Milano-Bicocca

Anticipated or actual start date

01 June 2014

Anticipated completion date

31 January 2015

Funding sources/ Sponsors

None

Endorsment

ESH Working Group on Cardiovascular Risk in Low Resource Settings
http://www.eshonline.org/Communities/WorkingGroups.aspx

Conflicts of interest

None known

Language

English

Countries

Italy, United Kingdom, The Netherlands,
