# Supplementary material for: Panethnic Differences in Blood Pressure in Europe: A Systematic Review and Meta-Analysis
Source: PLoS One. 2016 Jan 25;11(1):e0147601. doi: 10.1371/journal.pone.0147601 (PMC4725677; doi:10.1371/journal.pone.0147601)
Supplement: S1 Table — (PDF) [file pone.0147601.s003.pdf]

S1 Table. Influential analysis (Random effects model) for systolic blood pressure weighted mean difference (WMD)

**SA Women**	**Systolic WMD**	**95% Confidence Interval**		**p-value**	**τ^2**	**I^2**
Omitting McKeigue et al 1988	-2.73	-5.25	-0.22	0.0334	31.9328	96.20%
Omitting Cruickshank et al 1991	-2.90	-5.43	-0.37	0.0246	32.1188	96.20%
Omitting McKeigue et al 1991	-3.39	-5.90	-0.88	0.0082	31.2989	96.10%
Omitting Simmons et al 1993	-3.18	-5.83	-0.53	0.0187	34.9599	96.00%
Omitting Cappuccio et al 1998	-3.33	-5.86	-0.80	0.0099	31.636	96.10%
Omitting Bhopal et al 1999 (Indians)	-3.16	-5.70	-0.62	0.0149	32.1969	96.20%
Omitting Bhopal et al 1999 (Pakistanis)	-2.86	-5.40	-0.31	0.0277	32.2665	96.20%
Omitting Bhopal et al 1999 (Bangladeshi)	-2.57	-5.08	-0.05	0.0453	31.4473	96.10%
Omitting HSE 1999 (Indians)	-2.83	-5.43	-0.24	0.0324	33.4324	96.20%
Omitting HSE 1999 (Pakistanis)	-2.67	-5.19	-0.15	0.0375	31.3066	95.90%
Omitting HSE 1999 (Bangladeshis)	-2.53	-4.93	-0.13	0.0391	28.1792	95.40%
Omitting Whitty et al 1999	-3.10	-5.71	-0.49	0.0199	33.789	96.20%
Omitting HSE 2004 (Indians)	-2.78	-5.35	-0.22	0.0334	32.5896	96.20%
Omitting HSE 2004 (Pakistanis)	-2.67	-5.20	-0.15	0.0382	31.5528	96.00%
Omitting HSE 2004 (Bangladeshis)	-2.66	-5.18	-0.13	0.0394	31.6868	96.10%
Omitting Agyemang et al 2005	-3.63	-6.07	-1.20	0.0035	29.2692	95.80%
Omitting Lyratzopoulos et al 2005	-3.09	-5.64	-0.53	0.0181	32.5442	96.20%
Omitting Glenday et al 2006 (Pakistanis)	-2.97	-5.56	-0.38	0.0248	33.4295	96.20%
Omitting Glenday et al 2006 (Sri Lankians)	-2.82	-5.43	-0.20	0.0347	33.9688	96.10%
Omitting Gray et al 2011	-2.86	-5.48	-0.23	0.0328	34.2557	96.20%
Omitting Rabanal et al 2012	-3.15	-5.78	-0.52	0.0188	34.3707	96.10%
Omitting Agyemang et al 2015	-3.42	-5.69	-1.15	0.0032	24.9495	94.60%
**Pooled estimate**	**-2.97**	**-5.45**	**-0.49**	**0.019**	**31.9519**	**96.10%**

**SA Men**	**Systolic WMD**	**95% Confidence Interval**		**p-value**	**τ^2**	**I^2**
Omitting McKeigue et al 1988	-4.40	-6.06	-2.75	< 0.0001	15.5871	93.60%
Omitting Cruickshank et al 1991	-4.84	-6.48	-3.20	< 0.0001	15.4145	93.60%
Omitting McKeigue et al 1991	-4.98	-6.42	-3.54	< 0.0001	11.1813	90.80%
Omitting Knight et al 1992	-4.52	-6.19	-2.85	< 0.0001	15.8275	93.70%
Omitting Knight et al 1993	-4.54	-6.21	-2.87	< 0.0001	15.8854	93.70%
Omitting Simmons et al 1993	-4.64	-6.40	-2.89	< 0.0001	17.5556	93.70%
Omitting Cappuccio et al 1998	-4.86	-6.51	-3.22	< 0.0001	15.2829	93.50%
Omitting Bhopal et al 1999 (Indians)	-4.55	-6.21	-2.88	< 0.0001	15.8432	93.70%
Omitting Bhopal et al 1999 (Pakistanis)	-4.36	-6.02	-2.71	< 0.0001	15.4367	93.50%
Omitting Bhopal et al 1999 (Bangladeshi)	-4.17	-5.77	-2.56	< 0.0001	14.6001	93.20%
Omitting HSE 1999 (Indians)	-4.66	-6.37	-2.94	< 0.0001	16.6503	93.70%
Omitting HSE 1999 (Pakistanis)	-4.49	-6.18	-2.80	< 0.0001	16.1558	93.50%
Omitting HSE 1999 (Bangladeshis)	-4.36	-6.00	-2.72	< 0.0001	15.0396	93.20%
Omitting Whitty et al 1999	-4.69	-6.43	-2.94	< 0.0001	17.2491	93.60%
Omitting Lane 2002	-4.67	-6.35	-2.99	< 0.0001	16.0485	93.70%

Omitting HSE 2004 (Indians)	-4.59	-6.28	-2.90	< 0.0001	16.1624	93.70%
Omitting HSE 2004 (Pakistanis)	-4.46	-6.13	-2.79	< 0.0001	15.7946	93.60%
Omitting HSE 2004 (Bangladeshis)	-4.32	-5.95	-2.69	< 0.0001	14.9400	93.30%
Omitting Agyemang et al 2005	-4.80	-6.46	-3.14	< 0.0001	15.5890	93.60%
Omitting Lyratzopoulos et al 2005	-4.48	-6.16	-2.81	< 0.0001	15.8387	93.70%
Omitting Glenday et al 2006 (Pakistanis)	-4.59	-6.30	-2.89	< 0.0001	16.4800	93.70%
Omitting Glenday et al 2006 (Sri Lankians)	-4.46	-6.14	-2.77	< 0.0001	15.9751	93.20%
Omitting Gualdi-Russo et al 2009	-4.25	-5.85	-2.66	< 0.0001	14.1884	92.90%
Omitting Gray et al 2011	-4.63	-6.36	-2.91	< 0.0001	16.8865	93.70%
Omitting Rabanal et al 2012	-4.63	-6.38	-2.87	< 0.0001	17.5435	93.70%
Omitting Agyemang et al 2015	-4.81	-6.46	-3.16	< 0.0001	15.2481	93.00%
**Pooled estimate**	**-4.57**	**-6.20**	**-2.93**	**<0.0001**	**15.6804**	**93.50%**

**SSA Women**	**Systolic WMD**	**95% Confidence Interval**		**p-value**	**τ^2**	**I^2**
Omitting Meade 1978	5.96	2.04	9.87	0.0029	51.7041	96.10%
Omitting Sever 1979	5.39	1.52	9.25	0.0063	50.3579	96.00%
Omitting Haines 1987	6.73	2.89	10.58	0.0006	49.0332	95.60%
Omitting Cruickshank 1991	6.13	2.21	10.05	0.0022	51.7864	96.10%
Omitting Chaturvedi 1993	5.24	1.41	9.08	0.0074	48.9719	95.80%
Omitting Cappuccio 1998	5.66	1.68	9.65	0.0054	52.9141	95.90%
Omitting Whitty et al 1999	6.14	1.95	10.34	0.0041	59.0718	96.10%
Omitting HSE 1999	6.67	2.97	10.37	0.0004	44.8490	94.60%
Omitting Lane et al 2002	5.61	1.68	9.55	0.0051	51.5389	96.00%
Omitting HSE 2004 (BC)	6.67	2.79	10.55	0.0007	49.8003	95.60%
Omitting HSE 2004 (BA)	7.00	3.30	10.69	0.0002	44.8092	95.30%
Omitting Agyemang et al 2005	5.52	1.61	9.44	0.0057	50.8973	95.80%
Omitting Rabanal et al 2012	6.11	2.11	10.11	0.0027	53.4121	96.10%
Omitting Agyemang et al 2015 (AS)	5.75	1.62	9.87	0.0064	56.9091	95.50%
Omitting Agyemang et al 2015 (G)	5.47	1.60	9.34	0.0056	49.5323	95.50%
Omitting HSE 2004 (BA)	7.00	3.30	10.69	0.0002	44.8092	95.30%
**Pooled estimate**	**6.00**	**2.22**	**9.78**	**0.0018**	**50.9320**	**95.80%**

**SSA Men**	**Systolic WMD**	**95% Confidence Interval**		**p-value**	**τ^2**	**I^2**
Omitting Meade 1978	3.12	0.95	5.29	0.0048	16.5151	90.70%
Omitting Sever 1979	3.27	1.12	5.43	0.0029	16.5917	90.80%
Omitting Haines 1987	3.67	1.52	5.82	0.0008	16.1295	90.30%
Omitting Cruickshank 1991	3.16	1.02	5.30	0.0038	16.4043	90.80%
Omitting McKeigue 1991	3.12	0.90	5.34	0.0058	17.1805	89.70%
Omitting Chaturvedi 1993	3.24	1.07	5.42	0.0035	16.6996	90.80%
Omitting Cappuccio 1998	3.22	1.03	5.42	0.004	16.956	90.80%
Omitting Whitty 1999	3.47	1.15	5.78	0.0033	18.9189	90.60%
Omitting HSE 1999	3.65	1.50	5.80	0.0009	15.9677	89.50%

Omitting Lane et al 2002	3.22	1.00	5.43	0.0045	17.2515	90.70%
Omitting HSE 2004 (BC)	3.48	1.27	5.69	0.0021	17.1794	90.80%
Omitting HSE 2004 (BA)	3.80	1.70	5.89	0.0004	15.1327	89.80%
Omitting Agyemang et al 2005	3.09	0.92	5.26	0.0053	16.4854	90.50%
Omitting Gualdi-Russo et al 2009	4.00	2.03	5.96	< 0.0001	13.0346	88.40%
Omitting Rabanal et al 2012	3.73	1.60	5.85	0.0006	15.5467	89.90%
Omitting Agyemang et al 2015 (AS)	3.25	0.96	5.55	0.0054	18.5202	90.70%
Omitting Agyemang et al 2015 (G)	2.96	0.90	5.03	0.0049	14.4584	88.40%
**Pooled estimate**	**3.38**	**1.28**	**5.48**	**0.0016**	**16.3638**	**90.30%**
