# Supplementary material for: Panethnic Differences in Blood Pressure in Europe: A Systematic Review and Meta-Analysis
Source: PLoS One. 2016 Jan 25;11(1):e0147601. doi: 10.1371/journal.pone.0147601 (PMC4725677; doi:10.1371/journal.pone.0147601)
Supplement: S2 Table — (PDF) [file pone.0147601.s004.pdf]

S2 Table. Influential analysis (Random effects model) for Diastolic blood pressure weighted mean difference (WMD)

**SA Women**	**Diastolic WMD**	**95% Confidence Interval**		**p-value**	τ^2	I^2
Omitting McKeigue et al 1988	-0.08	-1.09	0.92	0.8696	4.4306	93.30%
Omitting Cruickshank et al 1991	0.10	-0.89	1.08	0.8449	4.2139	93.00%
Omitting McKeigue et al 1991	-0.19	-1.22	0.84	0.7208	4.5254	93.30%
Omitting Simmons et al 1993	-0.21	-1.29	0.87	0.7022	5.0417	93.20%
Omitting Cappuccio et al 1998	-0.27	-1.29	0.75	0.6011	4.4185	93.10%
Omitting Bhopal et al 1999 (Indians)	-0.12	-1.14	0.91	0.8237	4.4906	93.30%
Omitting Bhopal et al 1999 (Pakistanis)	-0.10	-1.12	0.92	0.8513	4.4824	93.30%
Omitting Bhopal et al 1999 (Bangladeshi)	-0.03	-1.05	0.98	0.9487	4.4211	93.30%
Omitting HSE 1999 (Indians)	-0.12	-1.18	0.94	0.8217	4.7896	93.30%
Omitting HSE 1999 (Pakistanis)	0.03	-0.97	1.02	0.9563	4.1351	92.30%
Omitting HSE 1999 (Bangladeshis)	-0.02	-1.05	1.00	0.9625	4.4345	92.90%
Omitting Whitty et al 1999	-0.20	-1.36	0.97	0.7380	5.9706	93.30%
Omitting HSE 2004 (Indians)	-0.17	-1.20	0.87	0.7535	4.5962	93.30%
Omitting HSE 2004 (Pakistanis)	-0.14	-1.17	0.90	0.7942	4.5566	93.30%
Omitting HSE 2004 (Bangladeshis)	-0.09	-1.12	0.93	0.8555	4.4725	93.30%
Omitting Agyemang et al 2005	-0.44	-1.40	0.53	0.3760	3.9034	92.40%
Omitting Lyratzopoulos et al 2005	-0.12	-1.14	0.91	0.8245	4.5035	93.30%
Omitting Glenday et al 2006 (Pakistanis)	-0.08	-1.12	0.95	0.8754	4.5475	93.30%
Omitting Glenday et al 2006 (Sri Lankians)	0.06	-0.91	1.03	0.9012	3.8845	91.80%
Omitting Gray et al 2011	-0.15	-1.21	0.92	0.7875	4.8278	93.30%
Omitting Agyemang et al 2015	-0.34	-1.18	0.50	0.4248	2.7374	88.40%
**Pooled estimate**	**-0.13**	**-1.12**	**0.87**	**0.8026**	**4.4210**	**93.00%**

**SA Men**	**Diastolic WMD**	**95% Confidence Interval**		**p-value**	τ^2	I^2
Omitting McKeigue et al 1988	-0.47	-1.64	0.69	0.4244	7.4947	94.60%
Omitting Cruickshank et al 1991	-0.58	-1.74	0.58	0.3285	7.5833	94.70%
Omitting McKeigue et al 1991	-0.77	-1.90	0.36	0.1833	6.9560	93.40%
Omitting Knight et al 1992	-0.58	-1.76	0.59	0.3289	7.6402	94.70%
Omitting Knight et al 1993	-0.62	-1.79	0.56	0.3037	7.6700	94.70%
Omitting Simmons et al 1993	-0.77	-1.91	0.37	0.1868	7.1337	93.70%
Omitting Cappuccio et al 1998	-0.73	-1.90	0.44	0.2236	7.6140	94.60%
Omitting Bhopal et al 1999 (Indians)	-0.36	-1.50	0.78	0.5340	7.2218	94.40%
Omitting Bhopal et al 1999 (Pakistanis)	-0.29	-1.41	0.82	0.6070	6.8404	94.10%
Omitting Bhopal et al 1999 (Bangladeshi)	-0.18	-1.26	0.91	0.7489	6.4019	93.70%
Omitting HSE 1999 (Indians)	-0.65	-1.86	0.56	0.2920	8.1228	94.70%
Omitting HSE 1999 (Pakistanis)	-0.46	-1.60	0.68	0.4303	7.1840	94.10%
Omitting HSE 1999 (Bangladeshis)	-0.48	-1.64	0.68	0.4175	7.3770	94.30%
Omitting Whitty et al 1999	-0.63	-1.86	0.60	0.3133	8.4332	94.70%
Omitting Lane 2002	-0.55	-1.73	0.62	0.3569	7.6545	94.60%
Omitting HSE 2004 (Indians)	-0.63	-1.81	0.55	0.2959	7.7609	94.70%

Omitting HSE 2004 (Pakistanis)	-0.57	-1.75	0.61	0.3427	7.6715	94.70%
Omitting HSE 2004 (Bangladeshis)	-0.50	-1.67	0.67	0.4014	7.5351	94.60%
Omitting Agyemang et al 2005	-0.78	-1.94	0.39	0.1902	7.4585	94.50%
Omitting Lyratzopoulos et al 2005	-0.48	-1.65	0.68	0.4162	7.4868	94.50%
Omitting Glenday et al 2006 (Pakistanis)	-0.68	-1.87	0.51	0.2601	7.8523	94.70%
Omitting Glenday et al 2006 (Sri Lankians)	-0.65	-1.87	0.57	0.2977	8.3026	94.70%
Omitting Gualdi-Russo et al 2009	-0.42	-1.57	0.73	0.4729	7.2629	94.40%
Omitting Gray et al 2011	-0.59	-1.80	0.62	0.3416	8.1142	94.60%
Omitting Agyemang et al 2015	-0.70	-1.92	0.51	0.2567	8.1821	94.60%
**Pooled estimate**	**-0.56**	**-1.71**	**0.58**	**0.3336**	**7.5473**	**94.50%**

**SSA Women**	**Diastolic WMD**	**95% Confidence Interval**		**p-value**	**τ^2**	**I^2**
Omitting Meade 1978	5.40	3.01	7.80	< 0.0001	17.5450	96.50%
Omitting Sever 1979	4.98	2.64	7.33	< 0.0001	17.1600	96.40%
Omitting Haines 1987	5.82	3.45	8.19	< 0.0001	16.8124	96.20%
Omitting Cruickshank 1991	5.31	2.91	7.71	< 0.0001	17.5565	96.50%
Omitting Chaturvedi 1993	4.92	2.55	7.29	< 0.0001	16.9454	96.30%
Omitting Cappuccio 1998	5.19	2.70	7.67	< 0.0001	18.6671	96.30%
Omitting Whitty et al 1999	5.52	3.05	7.99	< 0.0001	18.4211	96.40%
Omitting HSE 1999	5.76	3.73	7.79	< 0.0001	11.7872	93.40%
Omitting Lane et al 2002	5.12	2.70	7.53	< 0.0001	17.5999	96.40%
Omitting HSE 2004 (BC)	5.75	3.36	8.14	< 0.0001	17.1714	96.20%
Omitting HSE 2004 (BA)	5.81	3.44	8.18	< 0.0001	16.9119	96.20%
Omitting Agyemang et al 2005	5.10	2.66	7.53	< 0.0001	17.8195	96.30%
Omitting Agyemang et al 2015 (AS)	5.29	2.63	7.94	< 0.0001	21.5846	96.30%
Omitting Agyemang et al 2015 (G)	4.98	2.70	7.27	< 0.0001	15.4544	95.00%
**Pooled estimate**	**5.35**	**3.04**	**7.66**	**< 0.0001**	**17.3024**	**96.20%**

**SSA Men**	**Diastolic WMD**	**95% Confidence Interval**		**p-value**	**τ^2**	**I^2**
Omitting Meade 1978	5.40	3.01	7.80	< 0.0001	17.5450	96.50%
Omitting Sever 1979	4.98	2.64	7.33	< 0.0001	17.1600	96.40%
Omitting Haines 1987	5.82	3.45	8.19	< 0.0001	16.8124	96.20%
Omitting Cruickshank 1991	5.31	2.91	7.71	< 0.0001	17.5565	96.50%
Omitting Chaturvedi 1993	4.92	2.55	7.29	< 0.0001	16.9454	96.30%
Omitting Cappuccio 1998	5.19	2.70	7.67	< 0.0001	18.6671	96.30%
Omitting Whitty et al 1999	5.52	3.05	7.99	< 0.0001	18.4211	96.40%
Omitting HSE 1999	5.76	3.73	7.79	< 0.0001	11.7872	93.40%
Omitting Lane et al 2002	5.12	2.70	7.53	< 0.0001	17.5999	96.40%
Omitting HSE 2004 (BC)	5.75	3.36	8.14	< 0.0001	17.1714	96.20%
Omitting HSE 2004 (BA)	5.81	3.44	8.18	< 0.0001	16.9119	96.20%

Omitting Agyemang et al 2005	5.10	2.66	7.53	< 0.0001	17.8195	96.30%
Omitting Agyemang et al 2015 (AS)	5.29	2.63	7.94	< 0.0001	21.5846	96.30%
Omitting Agyemang et al 2015 (G)	4.98	2.70	7.27	< 0.0001	15.4544	95.00%
**Pooled estimate**	**5.35**	**3.04**	**7.66**	**< 0.0001**	**17.3024**	**96.20%**
