# Supplementary material for: Panethnic Differences in Blood Pressure in Europe: A Systematic Review and Meta-Analysis
Source: PLoS One. 2016 Jan 25;11(1):e0147601. doi: 10.1371/journal.pone.0147601 (PMC4725677; doi:10.1371/journal.pone.0147601)
Supplement: S3 Table — (PDF) [file pone.0147601.s005.pdf]

S3 Table. Effect of study quality on estimates of systolic (A) and diastolic (B) blood pressure (random effect metaregression)

A.

Variables	Point Estimate	95% CIs	p-value	Multiplicity adjusted p-value
		Men (n=43)		
**Panethnicity** (SSA vs. SA)	8.051	4.879 to 11.223	<0.001	<0.001
**NOS** (1 unit increase)	0.213	-1.497 to 1.922	0.730	0.900
		Women (n=37)		
**Panethnicity** (SSA vs. SA)	8.516	3.819 to 13.212	<0.001	0.001
**NOS** (1 unit increase)	-1.345	-4.432 to 1.742	0.393	0.610

SSA= Sub-Saharan Africans; SA = South Asians; EU = Europeans.

NOS = Newcastle-Ottawa Quality Score

B.

Variables	Point Estimate	95% CIs	p-value	Multiplicity adjusted p-value
		Men (n=41)		
**Panethnicity** (SSA vs. SA)	3.974	1.814 to 6.134	<0.001	<0.001
**NOS** (1 unit increase)	0.226	-0.926 to 1.379	0.701	0.940
		Women (n=35)		
**Panethnicity** (SSA vs. SA)	5.474	3.007 to 7.941	<0.001	<0.001
**NOS** (1 unit increase)	-0.104	-1.823 to 1.615	0.906	0.980

SSA= Sub-Saharan Africans; SA = South Asians; EU = Europeans.

NOS = Newcastle-Ottawa Quality Score
