# Supplementary figures and images for: Panethnic Differences in Blood Pressure in Europe: A Systematic Review and Meta-Analysis
Source: PLoS One. 2016 Jan 25;11(1):e0147601. doi: 10.1371/journal.pone.0147601 (PMC4725677; doi:10.1371/journal.pone.0147601)

# South Asians

## Author - Year

Systolic BP  
Weighted Mean  
Difference  
(95% CI)

### MEN

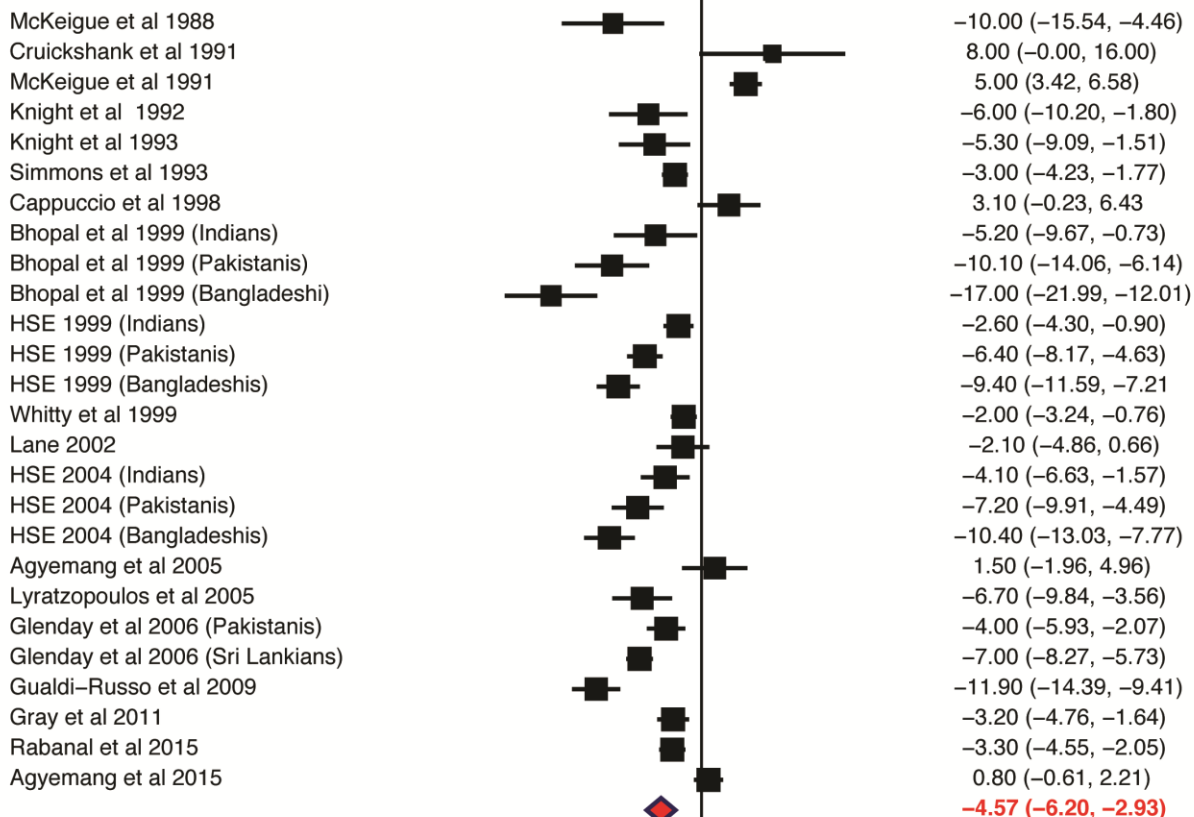

### WOMEN

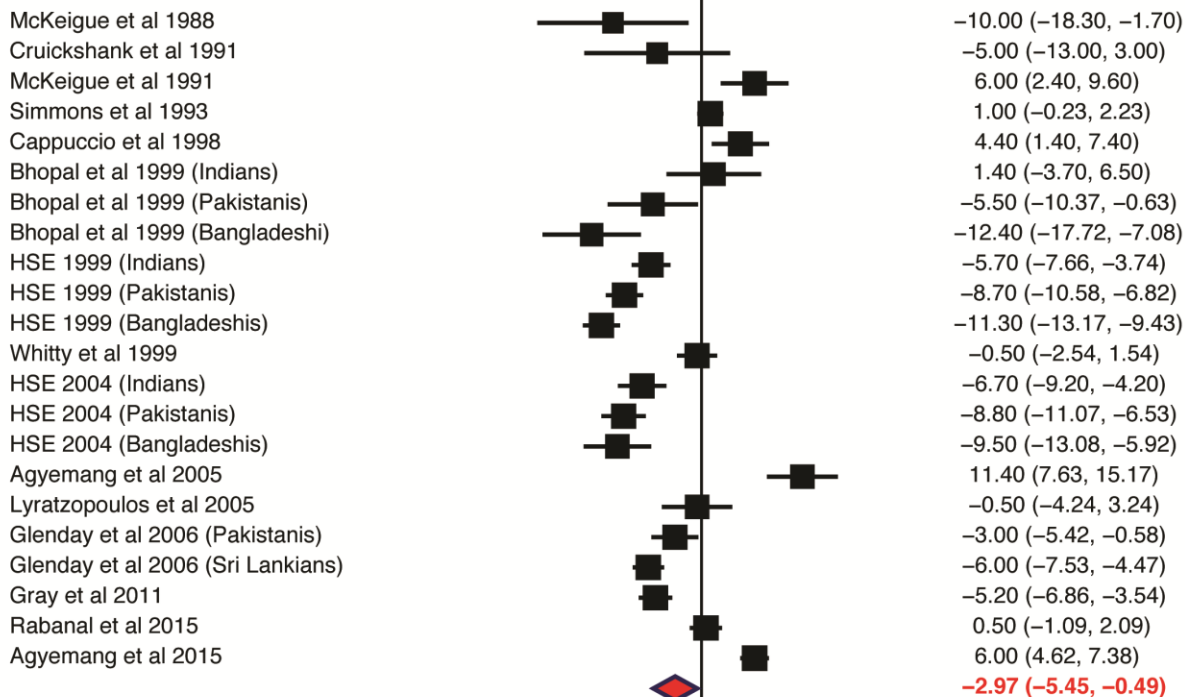

-10 -5 0 5 10  
mmHg

Supplement: S1 Fig — Forest plot of Systolic Blood Pressure mean differences between South Asians and Europeans. Estimates (95% CIs) are denoted by black boxes (black lines). A red diamond represents the pooled estimates for men and women, where diamond width corresponds to 95% CI bounds, “n” is the number of comparisons available within each subgroup. (PDF) [file pone.0147601.s006.pdf]

# South Asians

Author - Year

Diastolic BP  
Weighted Mean  
Difference  
(95% CI)

## MEN

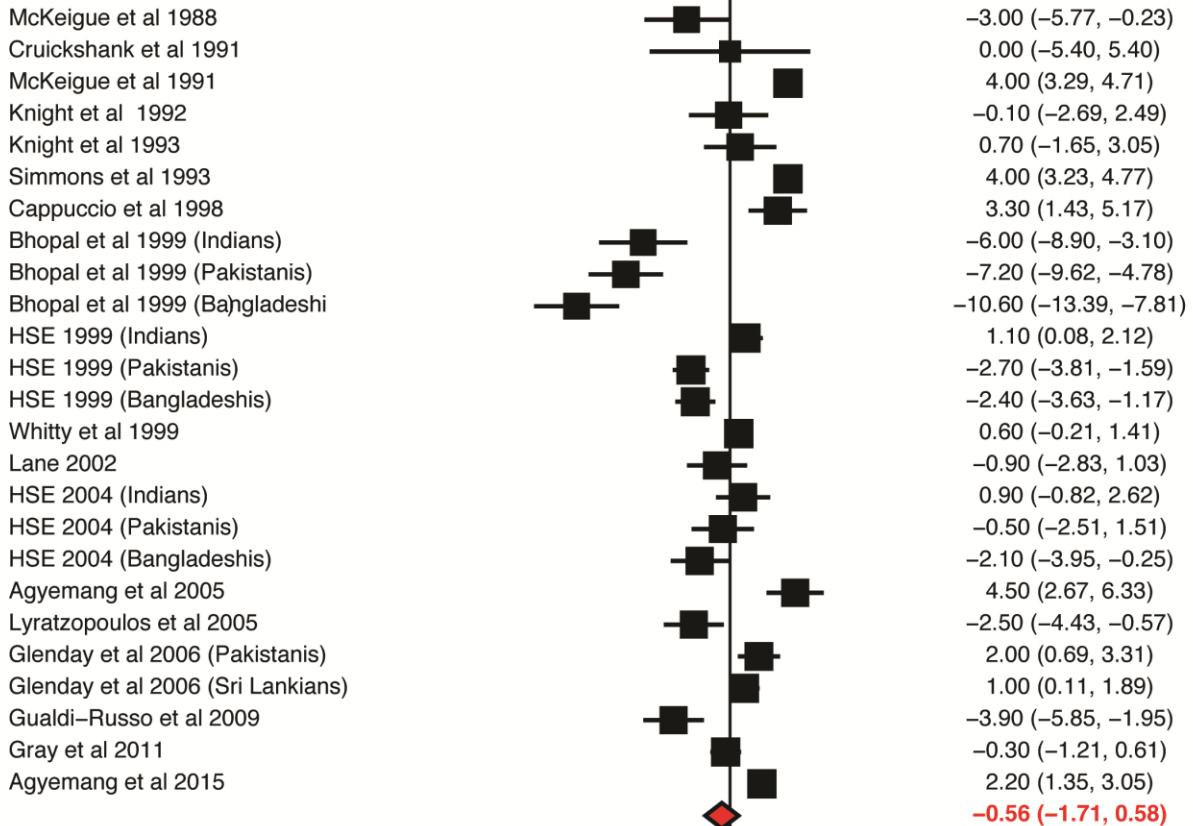

## WOMEN

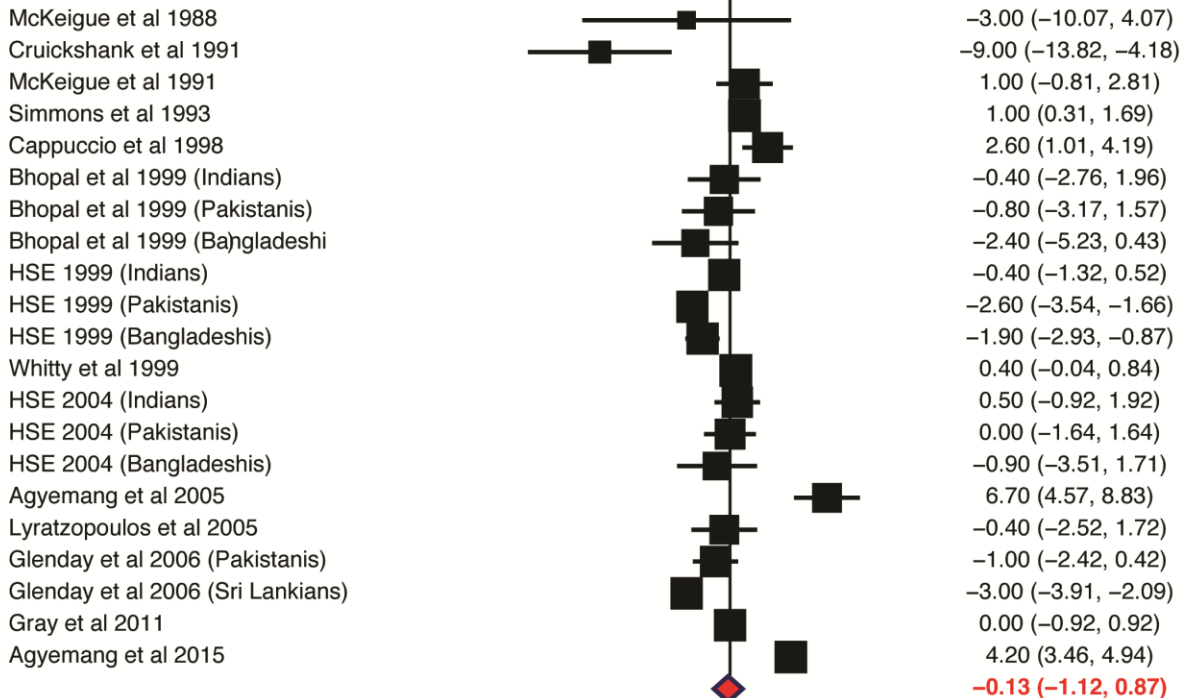

mmHg

Supplement: S2 Fig — Forest plot of Diastolic Blood Pressure mean differences between South Asians and Europeans. Estimates (95% CIs) are denoted by black boxes (black lines). A red diamond represents the pooled estimates for men and women, where diamond width corresponds to 95% CI bounds, “n” is the number of comparisons available within each subgroup. (PDF) [file pone.0147601.s007.pdf]

# Sub Saharan Africans

Author - Year

Systolic BP  
Weighted Mean  
Difference  
(95% CI)

## MEN

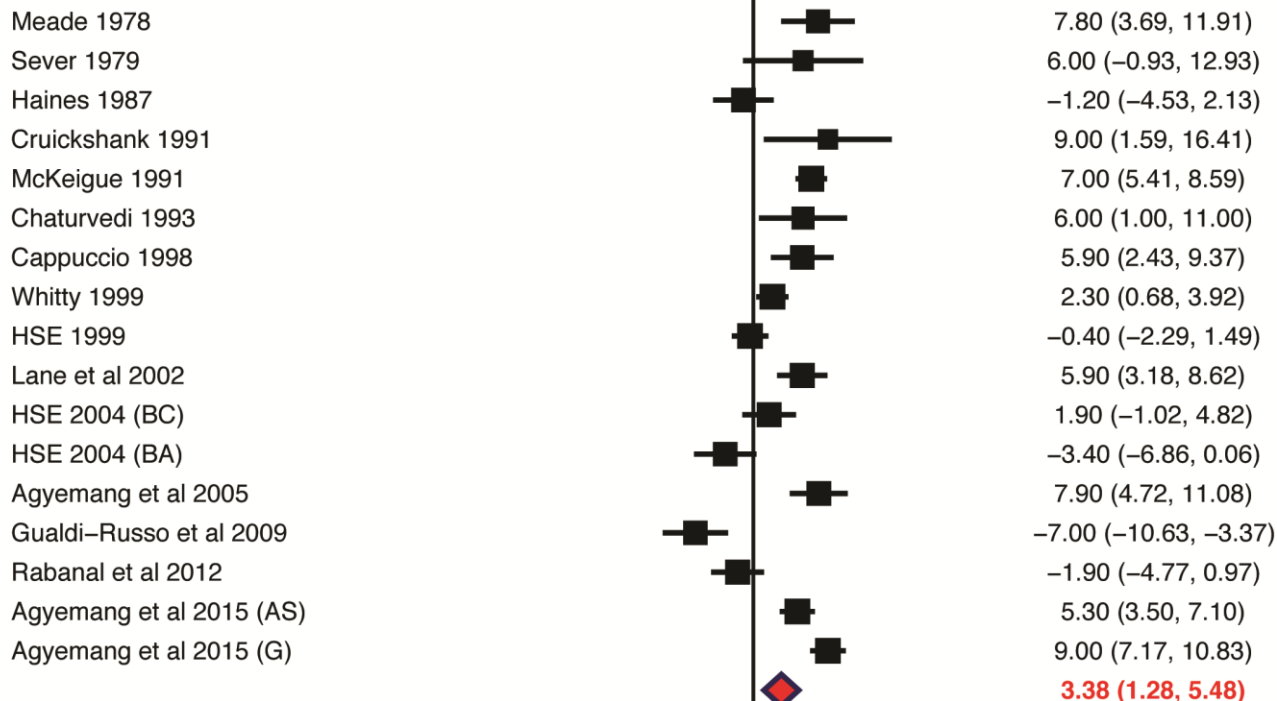

## WOMEN

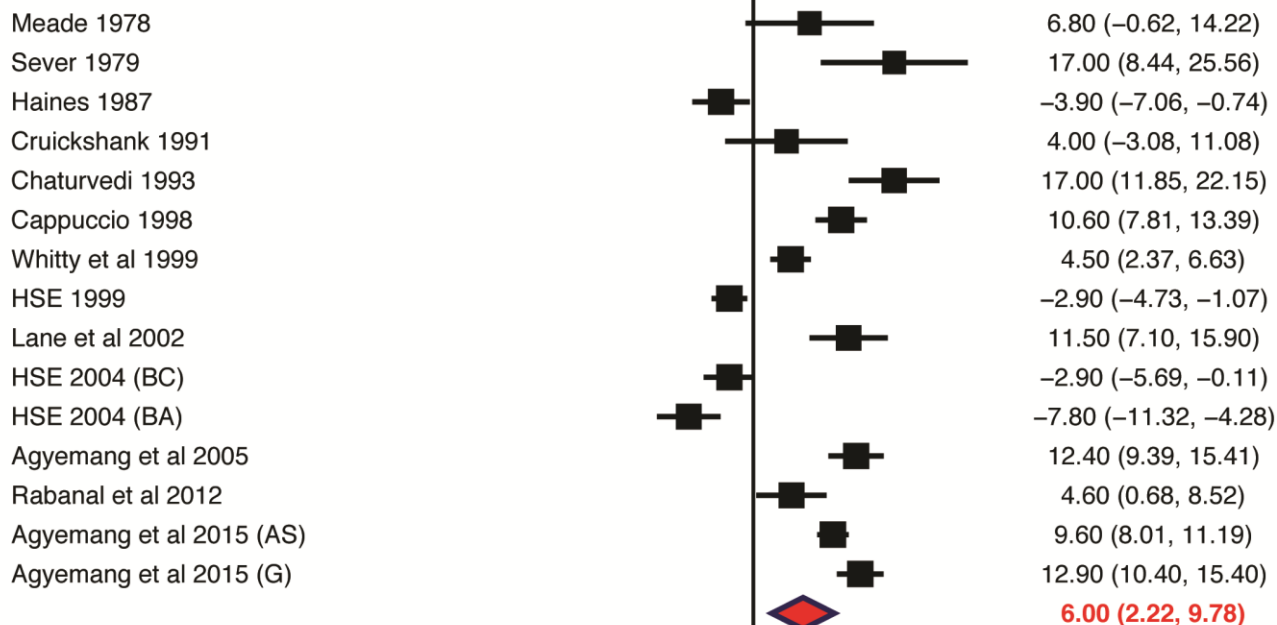

-10 -5 0 5 10  
mmHg

Supplement: S3 Fig — Forest plot of Systolic Blood Pressure mean differences between Sub Saharan Africans and Europeans. Estimates (95% CIs) are denoted by black boxes (black lines). A red diamond represents the pooled estimates for men and women, where diamond width corresponds to 95% CI bounds, “n” is the number of comparisons available within each subgroup. (PDF) [file pone.0147601.s008.pdf]

# Sub Saharan Africans

Author - Year

Diastolic BP  
Weighted Mean  
Difference  
(95% CI)

## MEN

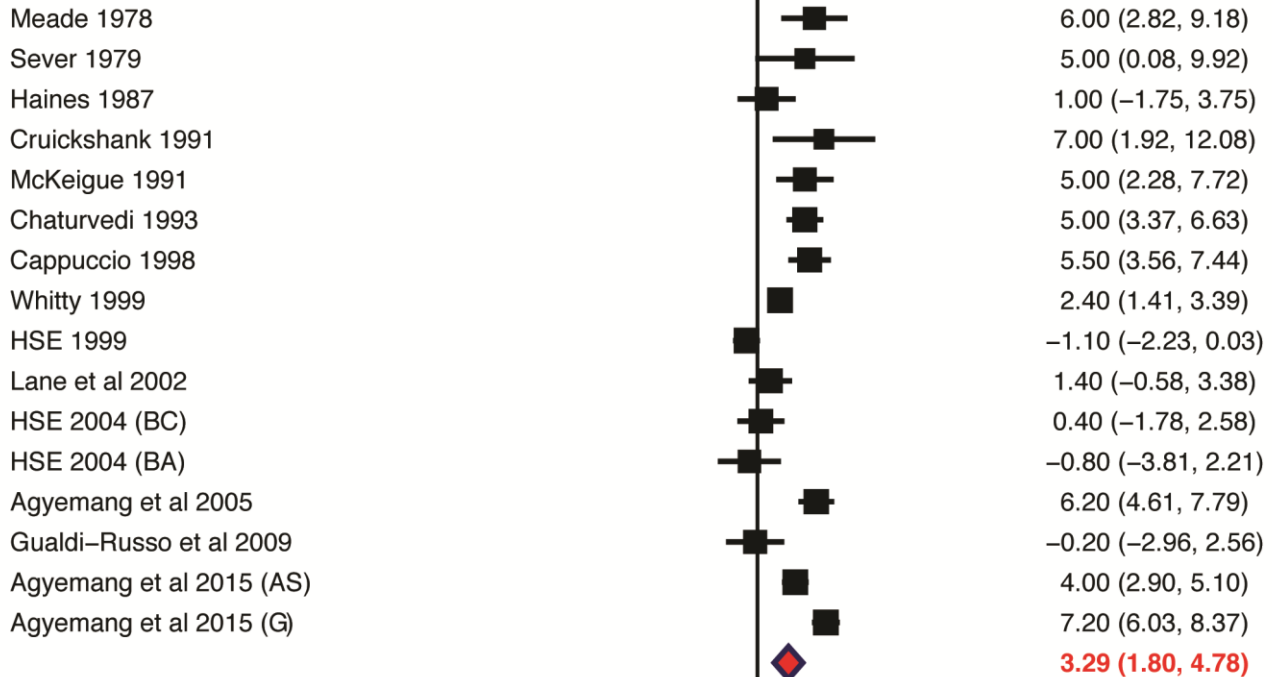

## WOMEN

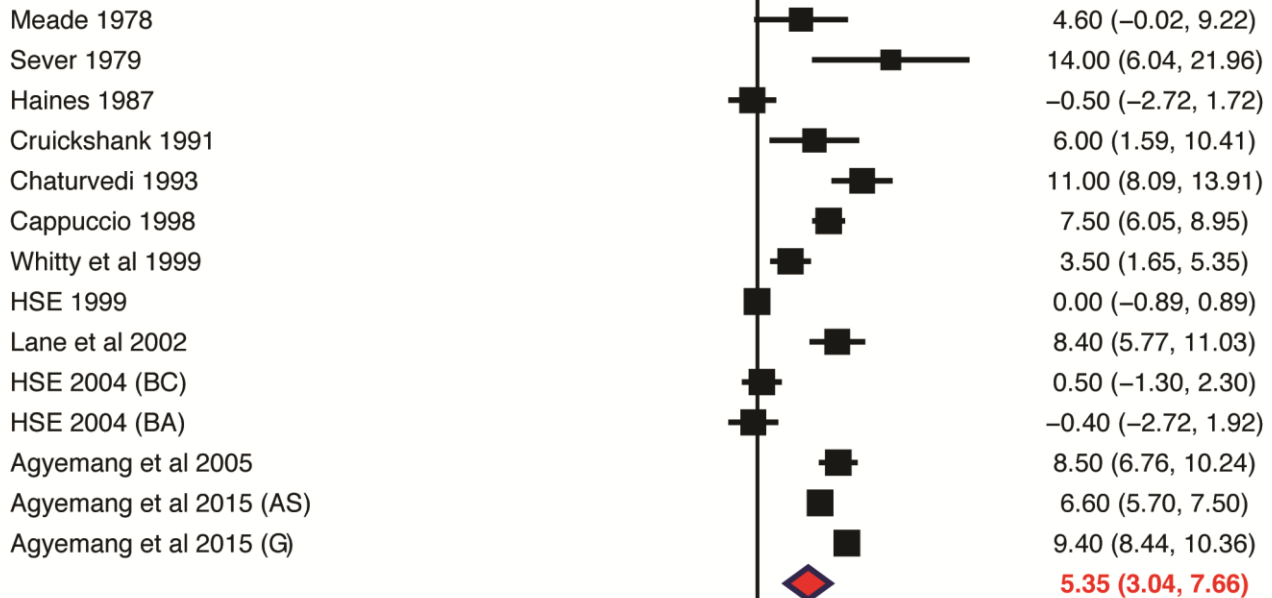

-10 -5 0 5 10  
mmHg

Supplement: S4 Fig — Forest plot of Diastolic Blood Pressure mean differences between Sub Saharan Africans and Europeans. Estimates (95% CIs) are denoted by black boxes (black lines). A red diamond represents the pooled estimates for men and women, where diamond width corresponds to 95% CI bounds, “n” is the number of comparisons available within each subgroup. (PDF) [file pone.0147601.s009.pdf]

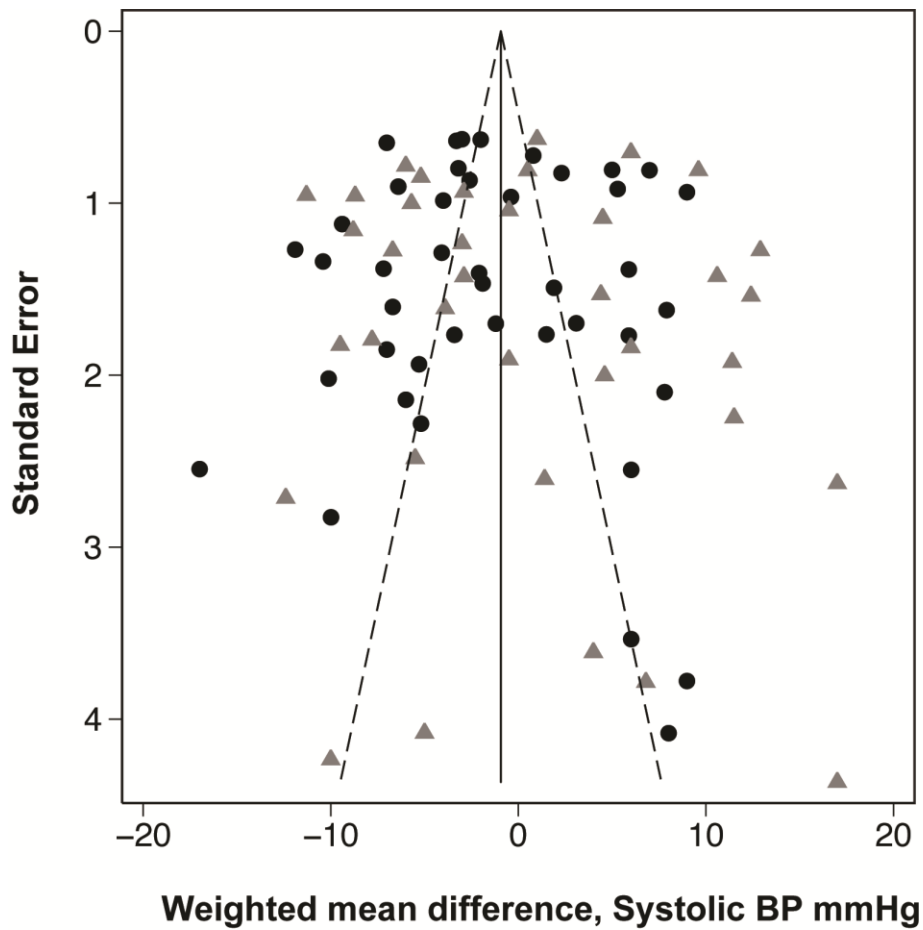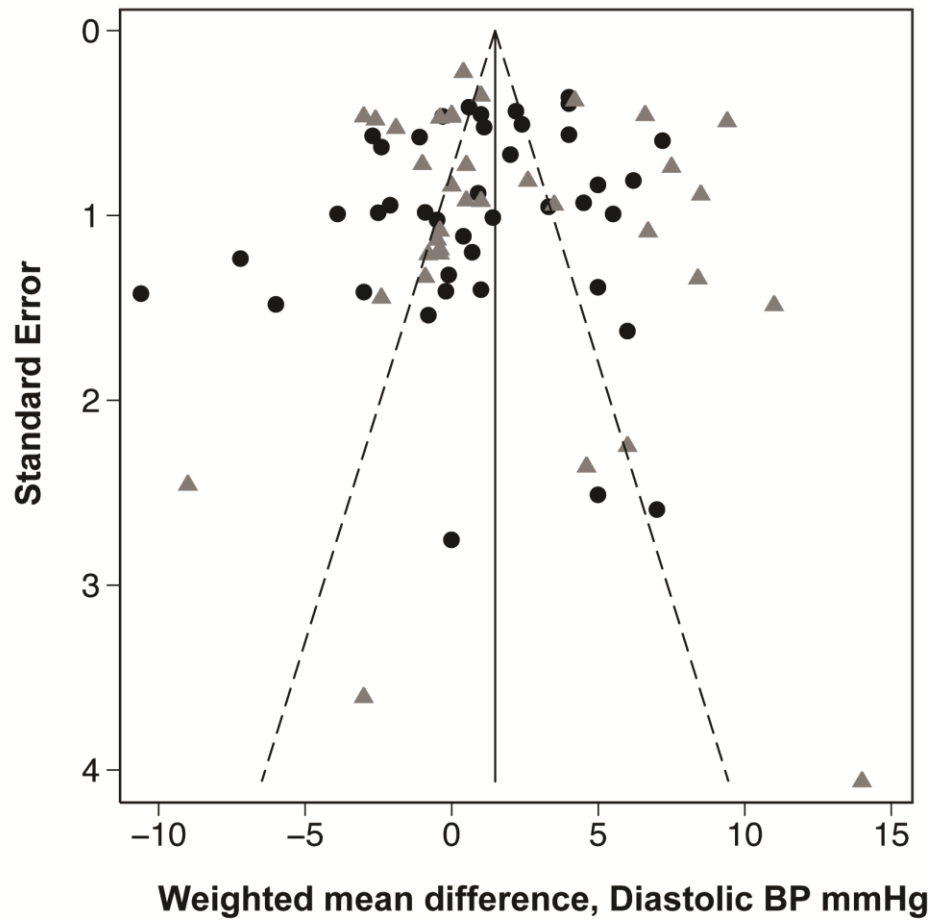

▲ Women ● Men

Supplement: S5 Fig — Funnel plots for publication bias. Each comparison is plotted by its effect size on the horizontal axis and its precision on the vertical axis. (PDF) [file pone.0147601.s010.pdf]

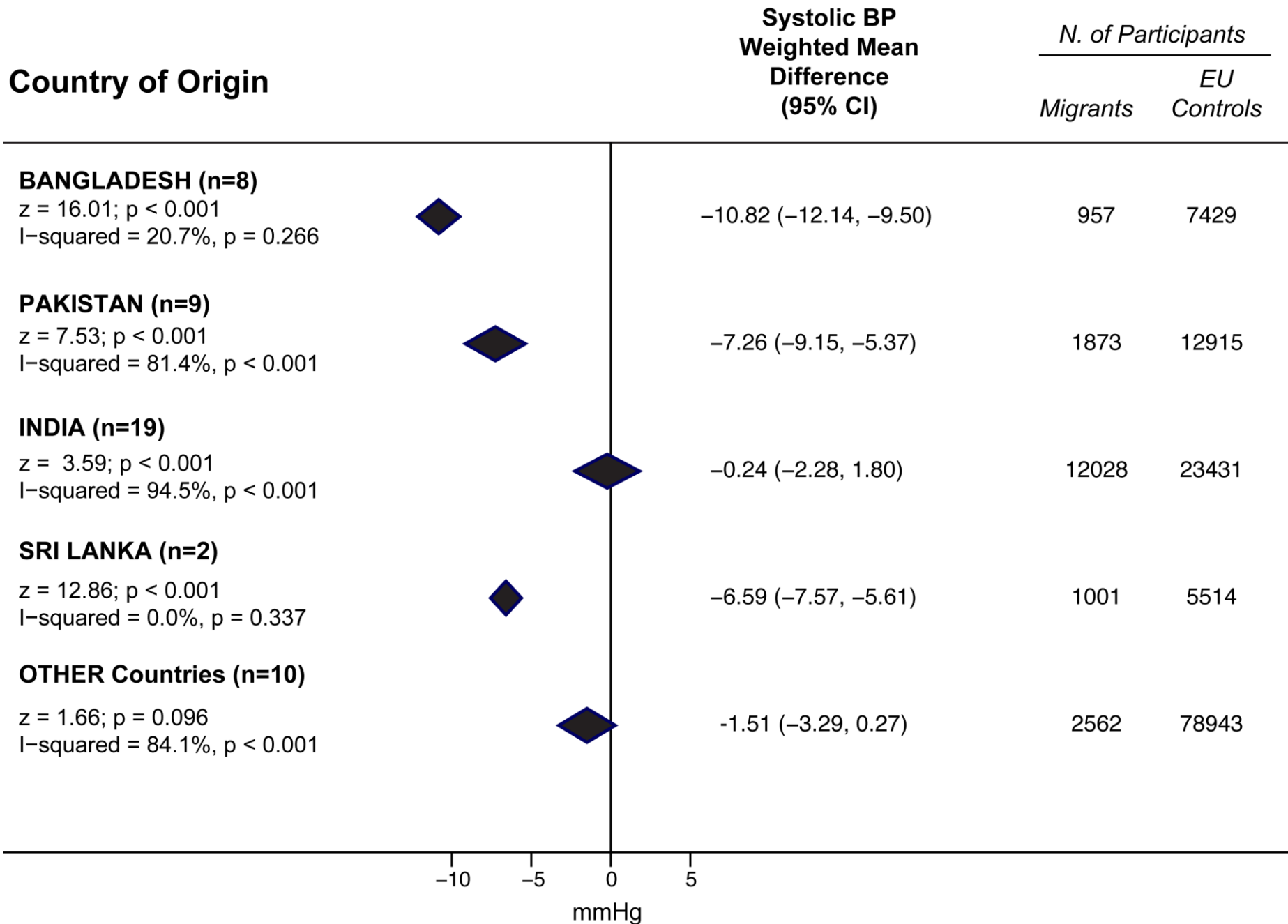

Supplement: S6 Fig — Subgroup comparisons of the weighted mean difference of systolic blood pressure between South Asians (SA) and European participants (EU) by country of origin. The term "others" refers to comparisons where the Country of origin of SA subjects was not specified. Diamonds denote the pooled estimates and 95% confidence intervals, “n” is the number of comparisons available within each subgroup. (PDF) [file pone.0147601.s011.pdf]

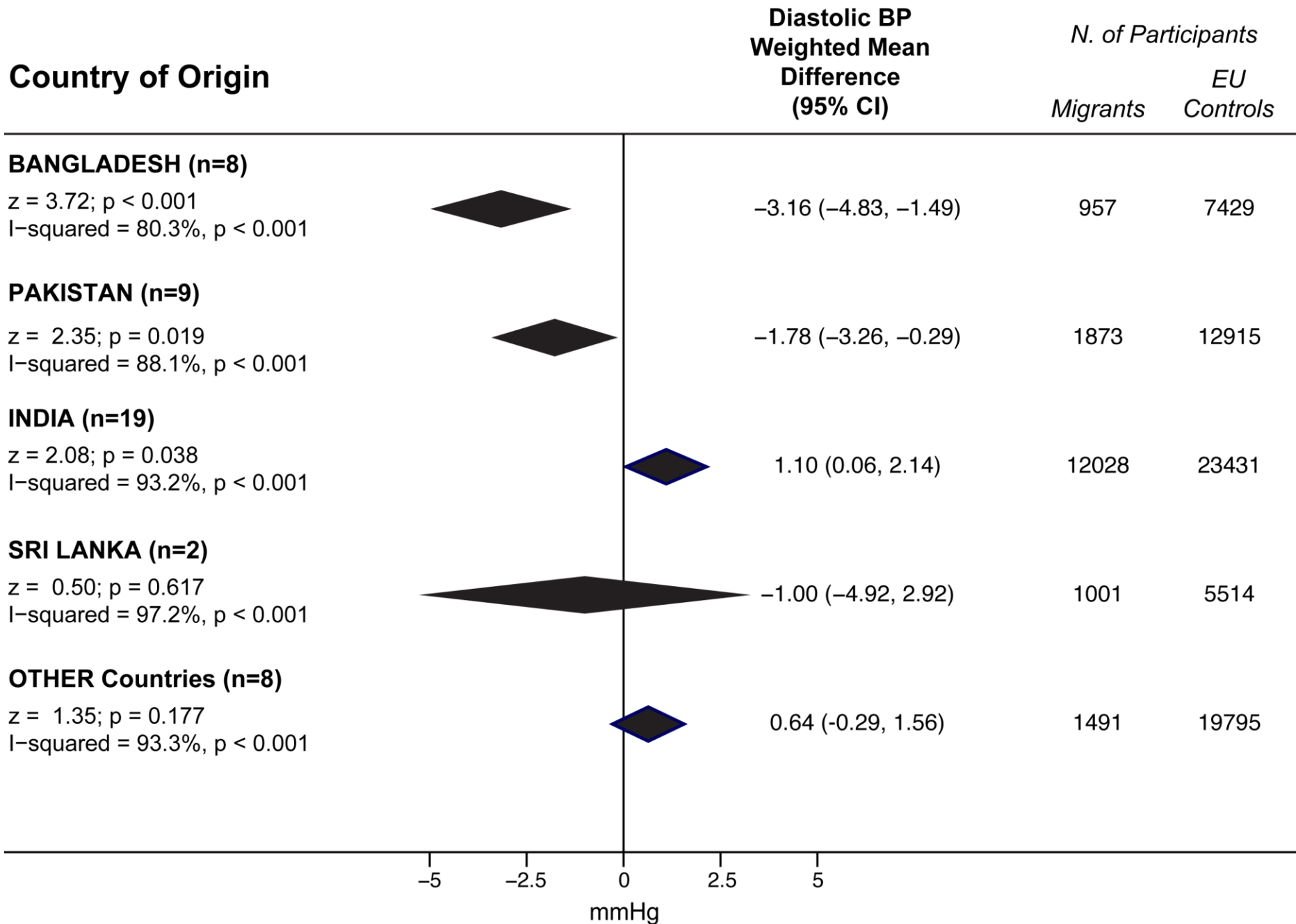

Supplement: S7 Fig — Subgroup comparisons of the weighted mean difference of diastolic blood pressure between South Asians (SA) and European participants (EU) by country of origin. The term "others" refers to comparisons where the Country of origin of SA subjects was not specified. Diamonds denote the pooled estimates and 95% confidence intervals, “n” is the number of comparisons available within each subgroup. (PDF) [file pone.0147601.s012.pdf]
