# Supplementary material for: Panethnic Differences in Blood Pressure in Europe: A Systematic Review and Meta-Analysis
Source: PLoS One. 2016 Jan 25;11(1):e0147601. doi: 10.1371/journal.pone.0147601 (PMC4725677; doi:10.1371/journal.pone.0147601)
Supplement: S8 Fig — Precision is on the horizontal axis plotted against the individual standardized effect sizes (vᵢ is the sampling variance of the observed effect size (ES) and τ² is the amount of heterogeneity, see methods for details). The central solid line represents the overall effect. Potential outliers (open symbols) lie either above or below the upper and lower confidence limits (dotted lines). (PDF) [file pone.0147601.s013.pdf]

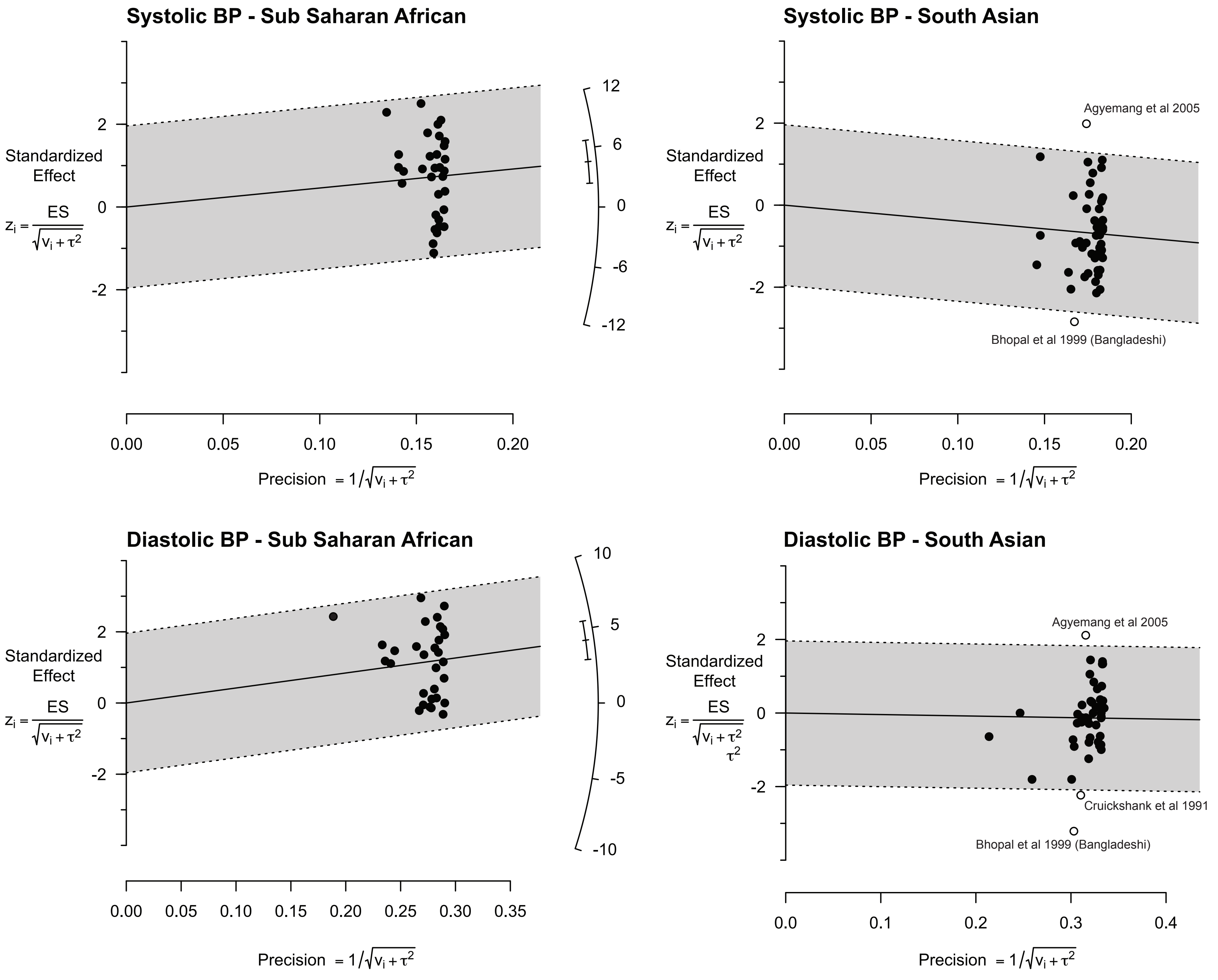
Systolic BP - Sub Saharan African
Systolic BP - South Asian
Diastolic BP - Sub Saharan African
Diastolic BP - South Asian
Standardized Effect
$z_i = \frac{ES}{\sqrt{v_i + \tau^2}}$
Precision $= 1/\sqrt{v_i + \tau^2}$
Agyemang et al 2005
Bhopal et al 1999 (Bangladeshi)
Cruickshank et al 1991
